# Photoneuroendocrine, circadian and seasonal systems: from photoneuroendocrinology to circadian biology and medicine

**DOI:** 10.1007/s00441-024-03913-7

**Published:** 2024-09-12

**Authors:** Horst-Werner Korf

**Affiliations:** Institute Anatomy I, Medical Faculty, Heinrich Heine University, Duesseldorf, Federal Republic of Germany

**Keywords:** Pinealocytes, Photoreceptors, Molecular clockwork, Clock genes, Lateral septal organ, Circadian rhythms, Circannual rhythms

## Abstract

This contribution highlights the scientific development of two intertwined disciplines, photoneuroendocrinology and circadian biology. Photoneuroendocrinology has focused on nonvisual photoreceptors that translate light stimuli into neuroendocrine signals and serve rhythm entrainment. Nonvisual photoreceptors first described in the pineal complex and brain of nonmammalian species are luminance detectors. In the pineal, they control the formation of melatonin, the highly conserved hormone of darkness which is synthesized night by night. Pinealocytes endowed with both photoreceptive and neuroendocrine capacities function as “photoneuroendocrine cells.” In adult mammals, nonvisual photoreceptors controlling pineal melatonin biosynthesis and pupillary reflexes are absent from the pineal and brain and occur only in the inner layer of the retina. Encephalic photoreceptors regulate seasonal rhythms, such as the reproductive cycle. They are concentrated in circumventricular organs, the lateral septal organ and the paraventricular organ, and represent cerebrospinal fluid contacting neurons. Nonvisual photoreceptors employ different photopigments such as melanopsin, pinopsin, parapinopsin, neuropsin, and vertebrate ancient opsin. After identification of clock genes and molecular clockwork, circadian biology became cutting-edge research with a focus on rhythm generation. Molecular clockworks tick in every nucleated cell and, as shown in mammals, they drive the expression of more than 3000 genes and are of overall importance for regulation of cell proliferation and metabolism. The mammalian circadian system is hierarchically organized; the central rhythm generator is located in the suprachiasmatic nuclei which entrain peripheral circadian oscillators via multiple neuronal and neuroendocrine pathways. Disrupted molecular clockworks may cause various diseases, and investigations of this interplay will establish a new discipline: circadian medicine.

## Introduction

To date, it is well known that biological rhythms play an important role in body functions (cf. Korf and von Gall 2024). This contribution aims to review the scientific development from photoneuroendocrinology to circadian biology which has unraveled the systemic, cellular, and molecular bases of biological rhythm entrainment and rhythm generation. Studies on color change mechanisms of a teleost fish, the European minnow (*Phoxinus phoxinus* L) performed by the later Nobel Prize Laureate Karl von Frisch in 1911 (von Frisch 1911), mark the beginning of a very fruitful research area on photoneuroendocrine, circadian, and circannual systems in the twentieth and twenty-first centuries. Von Frisch observed that shading of the head of blinded animals results in the contraction of melanophores, whereas illumination causes expansion of the melanin pigment. From these experiments, von Frisch concluded that the light-sensitive region eliciting this response is located within the brain (skull) and suspected it to be the pineal organ. Indeed, pinealectomy abolished the response for 1 day, but thereafter, the light-dependent reactivity of melanophores returned. These results led to the conclusion that the pineal organ of the minnow is an important but not the only site of extraocular photoreception. This conclusion is still valid; it applies to all nonmammalian species investigated to date and gave rise to the concept of multiple photoreceptor systems which are located in the retina, the pineal complex, and deep in the brain (“deep brain photoreceptors” or “encephalic photoreceptors”) and serve different functions. Later, Karl von Frisch asked his student Ernst Scharrer to search for “deep brain photoreceptors.” By doing so, Ernst Scharrer performed remarkable studies with blinded minnows in which he discovered the “gland-like nature” of hypothalamic neurons (Scharrer 1928). Based on these investigations, Ernst Scharrer, his wife Berta Scharrer, and Wolfgang Bargmann founded the concept of neurosecretion and a new biomedical discipline: Neuroendocrinology (reviewed by Rodriguez et al. 2024).

**Fig. 1:**
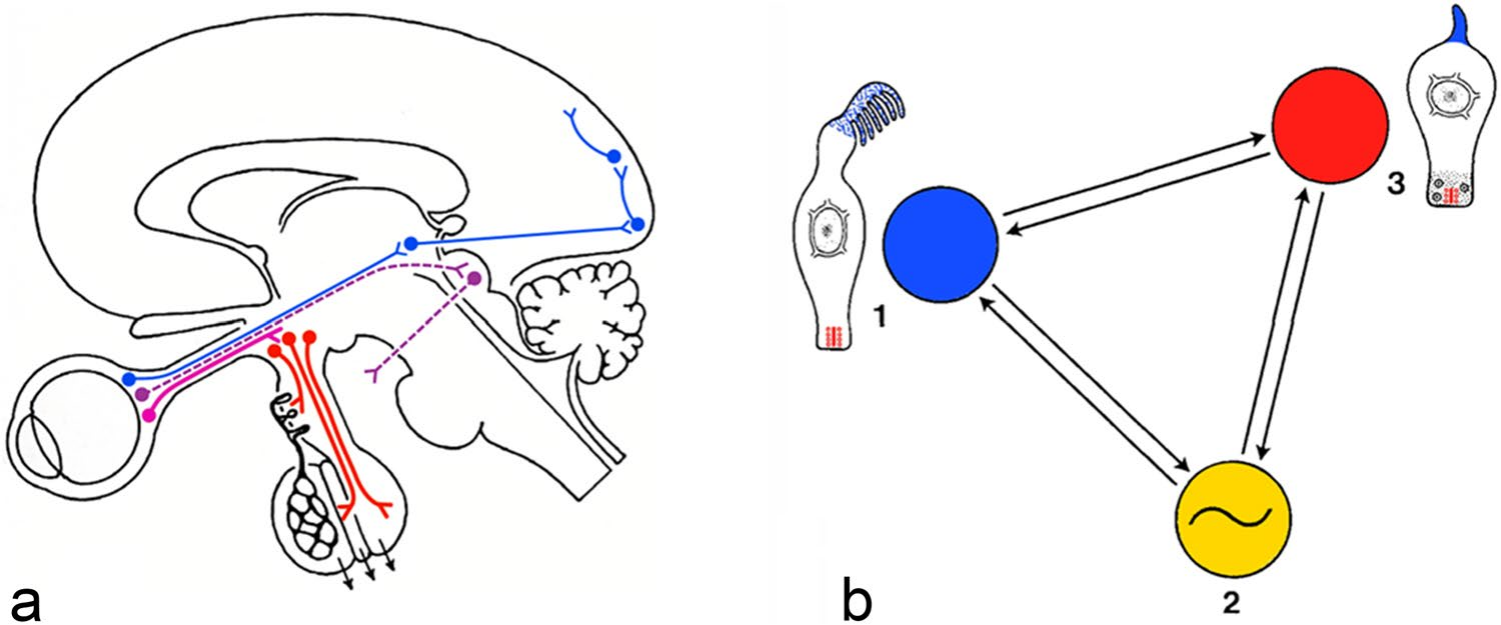
Photoneuroendocrine systems. **a** (blue) Visual pathway from the retina to the lateral geniculate to the occipital cortex; (purple) pathway for control of optic reflexes from the retina to the mesencephalon (pretectal area); (purple-red) photoneuroendocrine pathway with retinohypothalamic tract and hypothalamic neuroendocrine centers (Scharrer 1964). **b** Key elements of photoneuroendocine systems: (1) nonvisual photoreceptor as luminance detector, (2) endogenous circadian rhythm generator, (3) neuroendocrine effector. The key elements may be located in a single cell (photoneuroendocrine cell)

In 1964, Ernst Scharrer presented general concepts on photo-neuro-endocrine systems (E. Scharrer 1964) and pointed out that they serve the translation of photic stimuli into neuroendocrine responses. They are thus distinct from the visual system that transforms light pulses into synaptic responses and serves image analysis via neuronal mechanisms (Fig. 1).

While the visual system allows for spatial orientation, photoneuroendocrine systems control neuroendocrine functions thereby allowing to measure and keep the time. The input to the photoneuroendocrine system is provided by “nonvisual” or “circadian” photoreceptors.

In general, nonvisual (circadian) photoreceptors serve as luminance detectors and provide an important input to circadian and circannual systems by entraining them to the ambient lighting conditions that serve as “zeitgeber” (Aschoff et al. 1975). The majority of nonvisual photoreceptors contain photopigments with absorption maxima in the UV/blue range of the spectrum (Guido et al. 2022) and they occur rather early in ontogenetic development (Do and Yau 2010; Contin et al. 2006).

## The pineal complex as a component of the photoneuroendocrine system 

**Fig. 2:**
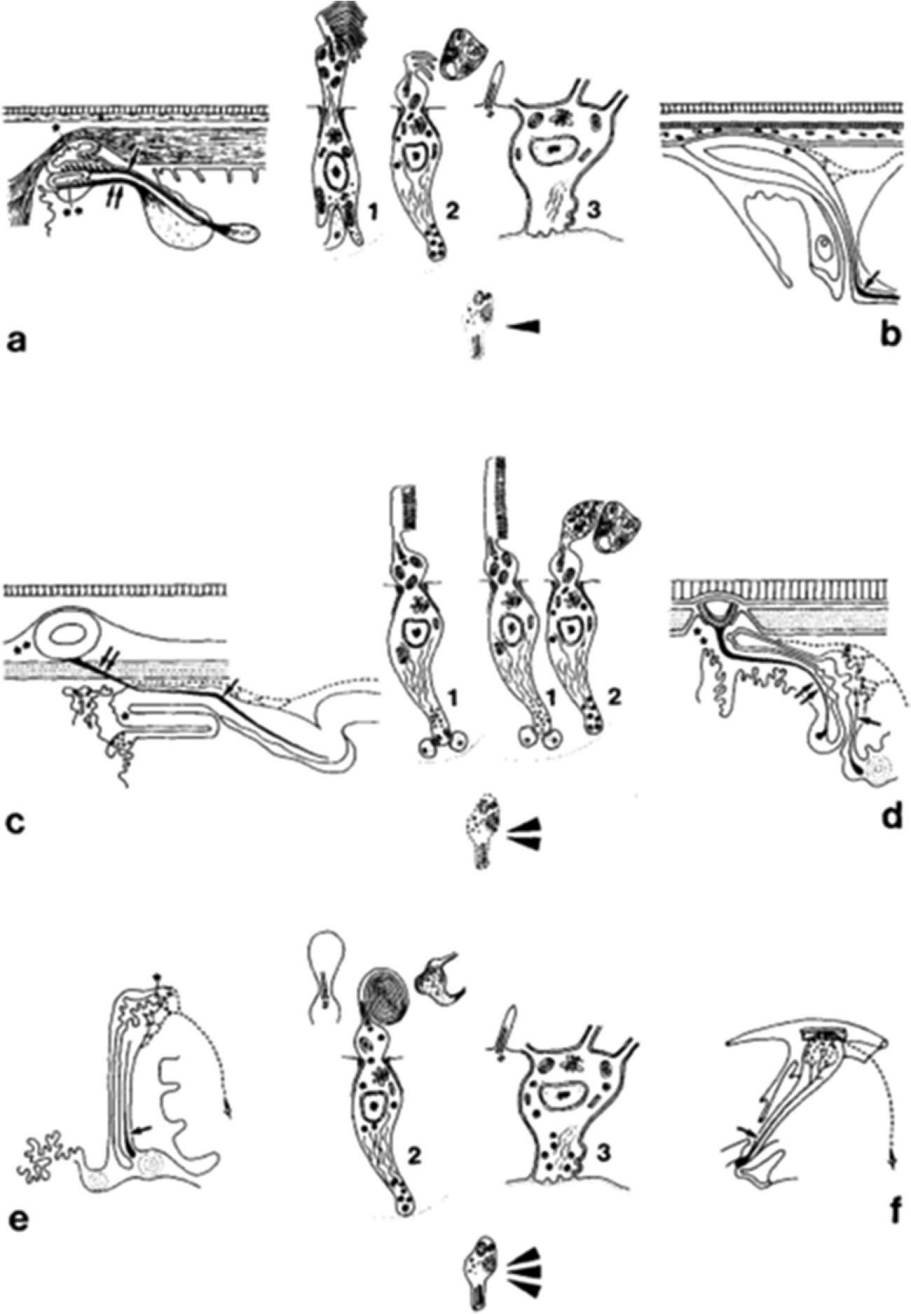
Phylogenetic transformation of the pineal complex. Macroscopic appearance of the pineal complex as seen in the sagittal plane and ultrastructure of pinealocytes of the sensory line in cyclostomes (**a**), teleosts (**b**), anurans (**c**), lacertilians (**d**), birds (**e**), and mammals (**f**). The pineal complex of cyclostomes and teleosts comprises a parapineal and a pineal organ; in anurans, the pineal complex is divided into an extracranial frontal organ and the pineal organ; lacertilians display an extracranial parietal eye and a pineal organ proper. Birds and mammals only possess an intracranial pineal organ. Dotted lines, noradrenergic (sympathetic) nerve fibers; arrows, central (pinealofugal) innervation; (1), true pineal photoreceptor with regularly lamellated outer segment and synaptic connections with second-order neurons giving rise to the pineal tract projecting to di- and mesencephalic brain regions; (2), modified pinealocyte with an irregular outer segment or bulbous cilium; (3), neuroendocrine pinealocyte of the mammalian type lacking an outer segment and the direct photosensitivity. In most vertebrate classes, the pineal complex comprises a circadian rhythm generator; in some teleosts (e.g., rainbow trout) and in all mammals, the circadian rhythm generator is absent from the pineal. The noradrenergic innervation develops progressively in the course of evolution. In teleosts and anurans, noradrenergic nerve fibers are only found in the capsule of the pineal. In reptiles, they penetrate into the pineal; in birds and mammals, they form a dense network within the pineal (modified after Korf 1994)

**Fig. 3:**
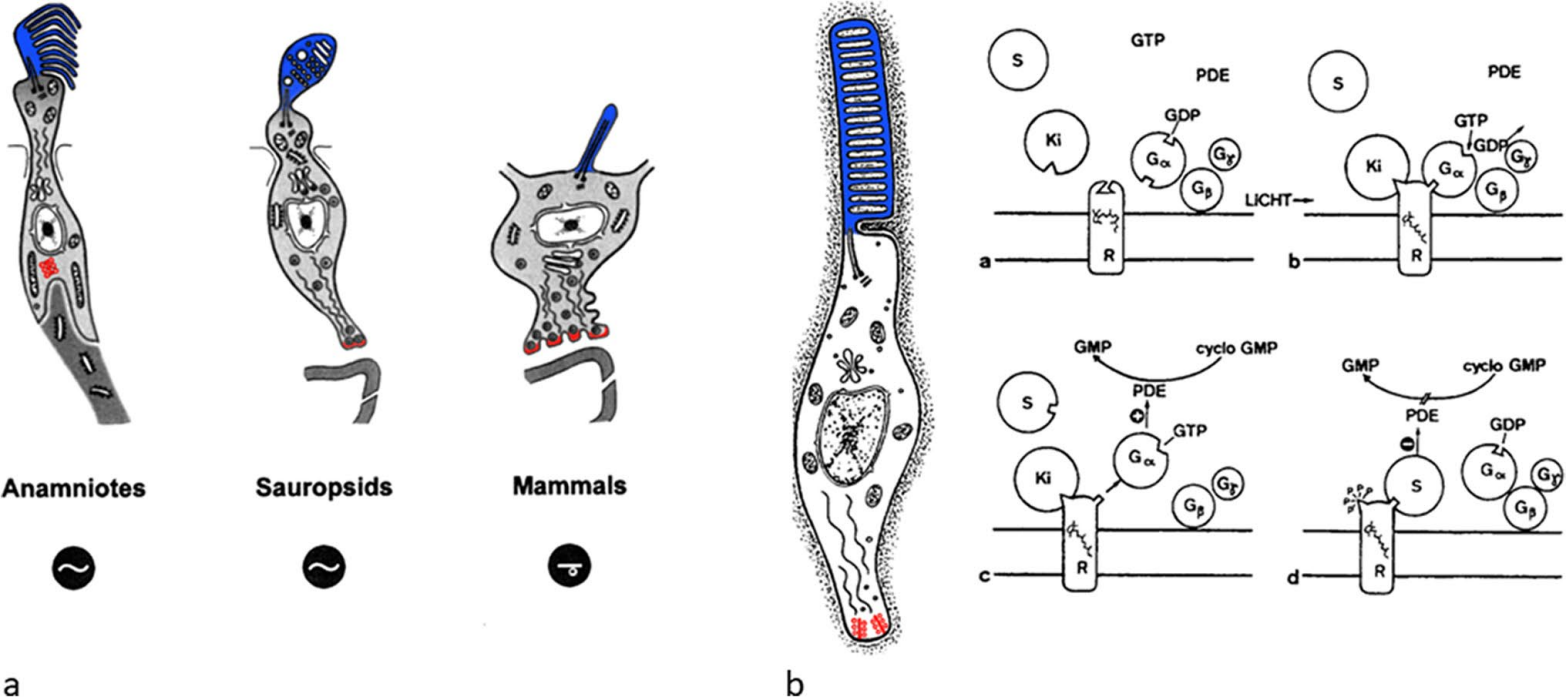
**a** Pinealocytes of the receptor line. True pineal photoreceptors bearing regularly lamellated outer segments (blue) and establishing synaptic contacts (red) with intrapineal second-order neurons. They predominate in anamniotes and may or may not have rhythm-generating capacities. Modified pineal photoreceptors predominate in sauropsids (reptiles and birds); they bear rudimentary outer segments or just a bulbous cilium (blue), retain direct light sensitivity, and have rhythm-generating capacities. Purely neuroendocrine pinealocytes occur in mammals. They have lost the direct light sensitivity and the rhythm-generating capacity. **b** Phototransduction cascade in retinal rods and photoreceptor-specific proteins. Rod-opsin with the prosthetic group, Galpha, beta, gamma subunits of transducin, PDE cyclic GMP phosphodiesterase, S S-antigen (arrestin), and Ki rhodopsin kinase

**Fig. 4:**
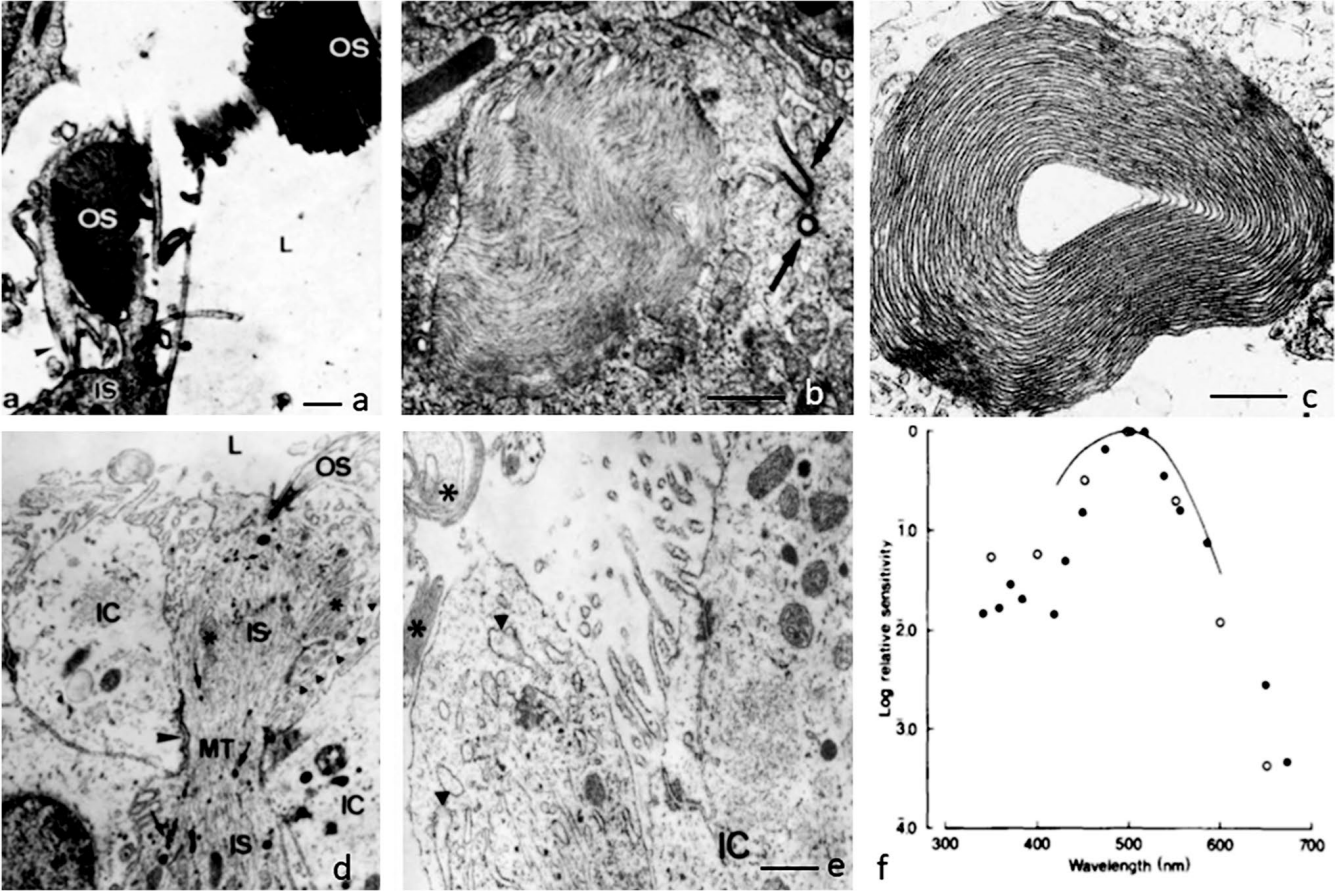
Ultrastructure of pinealocytes of the receptor line. **a**–**c** Regularly lamellated outer segments of true pineal photoreceptors of the tiger salamander, *Ambystoma tigrinum* (**a**, Korf 1976), and the clawed toad, *Xenopus laevis* (**b**, **c**, Korf et al. 1981). **d**, **e** Modified pineal photoreceptors with rudimentary outer segments of the agamid lizard, *Uromastix hardwickii* (Hafeez et al. 1987). OS outer segments, IS inner segments, MT microtubules, IC interstitial cell, L lumen of the pineal organ, arrowhead zonula adherens, arrow cilium of 9 + 0 type, asterisk ectopic membrane whorls as outer segment remainder, triangles dilated rough endoplasmic reticulum. Bars = 2 µm. **f** Relative spectral sensitivity of the chromatic response recorded from the frontal organ of *Xenopus laevis*: inhibitory action spectrum (open circles); excitatory action spectrum (dots). The light threshold of the excitatory component is 2.2 log units lower than the inhibitory threshold. The curve drawn in full represents Dartnall’s nomogram v.p. 520 nm (Dartnall 1953) (Korf et al. 1981)

**Fig. 5:**
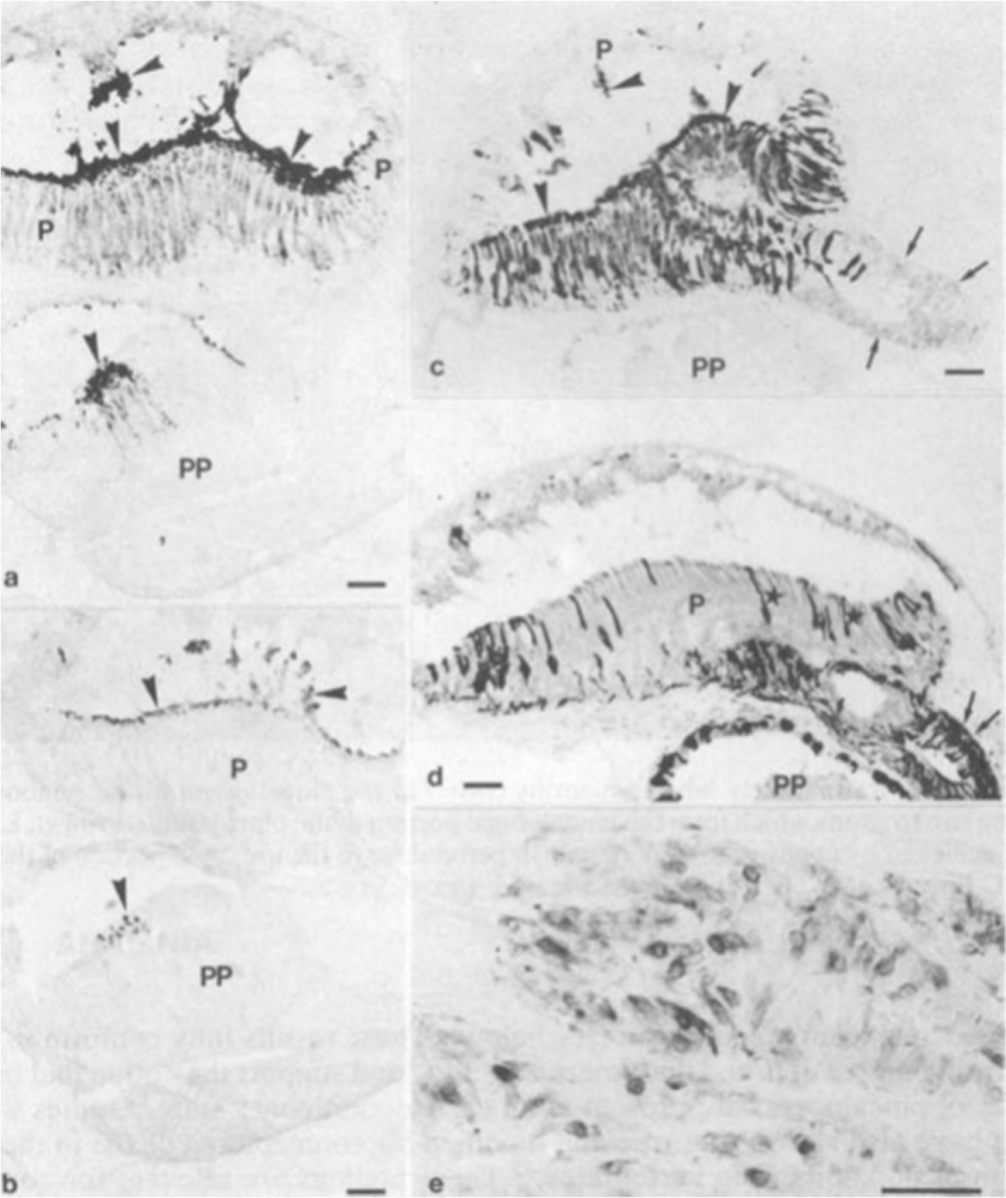
Immunocytochemical demonstration of photoreceptor-specific proteins and serotonin, the precursor of melatonin in pinealocytes of the lamprey, *Lampetra japonica*, and the mallard, *Anas platyrhynchos*. **a**–**d** Sagittal sections through the pineal (P) and parapineal organs (PP) of the lamprey. **a** Rod-opsin immunoreactive pineal photoreceptors. Intense labeling of outer segments (arrowheads), protruding into the pineal lumen. **b** alpha-transducin immunoreaction is restricted to the outer segments of the pineal photoreceptors. **c** Strong S-antigen immunoreaction is found in outer segments (arrowheads), perikarya, and processes of pineal photoreceptors. The proximal part of the pineal (atrium) contains cells resembling pinealocytes of the mammalian type; these display a weak S-antigen immunoreaction (arrows). **d** Serotonin-immunoreaction is found in bipolar cells resembling modified photoreceptors (stars) and in the pinealocytes of the mammalian type (arrows). **e** S-antigen immunoreaction in modified pineal photoreceptors of the mallard. Bars = 50 µm

**Fig. 6:**
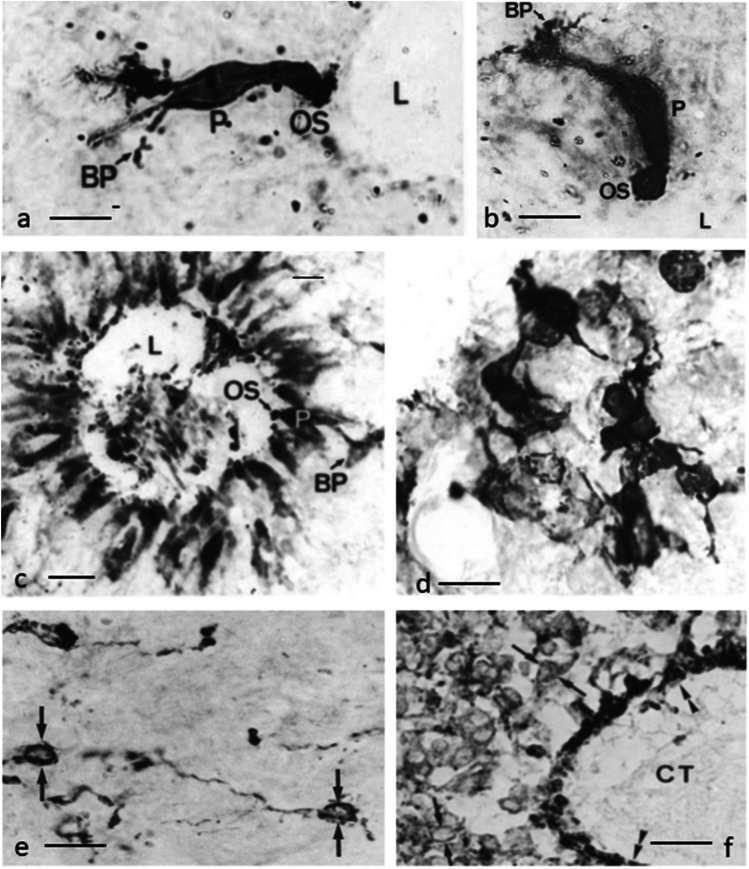
**a, b **Rod-opsin immunoreaction in modified pineal photoreceptors of the Japanese quail (Foster et al. 1987), **c**﻿ alpha-Transducin immunoreaction in numerous modified pineal photoreceptors of the Japanese quail (Foster et al. 1987), **d** rod-opsin immunoreaction in pinealocytes of C57/Bl mouse (Korf et al. 1985a), and e﻿ human (Huang et al. 1992). **f** Pinealocytes of the receptor line share neuronal characteristics. Strong synaptophysin immunoreactions are observed in numerous human pinealocytes. End feet of pinealocytes contacting the basal lamina are strongly labeled (double arrowheads), CT septa with connective tissue (Huang et al. 1992). Bar in a, b, d = 25 µm, in c = 40 µm, in e 20 µm, in f 30 µm

The pineal complex has undergone a remarkable transformation in the course of phylogeny. Being a directly light-sensitive organ in poikilothermic vertebrates, it serves as a purely neuroendocrine organ in mammals including man (Fig. 2). In parallel to this development, the pineal-specific cells, the pinealocytes appear as true pineal photoreceptors, modified pineal photoreceptors, and pinealocytes sensu stricto, all of which are believed to belong to a common cell lineage (Fig. 3). True pineal photoreceptors have been investigated by means of electron microscopy in teleosts (Oksche and Kirschstein 1967, 1971), anurans (Oksche and von Harnack 1963, Oksche and Vaupel-von Harnack 1963, Ueck 1968), urodeles (Korf 1976), lacertilians (Oksche and Kirschstein 1968) and modified pineal photoreceptors in reptiles (Hafeez et al. 1987) and birds (Oksche and Kirschstein 1969, Oksche and Vaupel-von Harnack 1966, Oksche et al. 1969, 1972) (Fig. 4). True pineal photoreceptors display synaptic ribbons in their basal processes and are synaptically connected to intrapineal second order neurons (see below). The modified pineal photoreceptors and mammalian pinealocytes display also synaptic ribbons and a varying number of dense core granules. Neurophysiological studies with directly light-sensitive pineal organs of fish and frogs have revealed photopigments with absorption maxima at 500 nm (Dodt and Heerd 1962; Morita 1966). In the clawed toad, *Xenopus laevis*, two types of responses to light stimuli, achromatic and chromatic, were recorded from the frontal organ, while the epiphysis (pineal organ proper) exhibited only achromatic units. The opposed color mechanism of the chromatic response showed a maximum sensitivity at approximately 360 nm for the inhibitory and at 520 nm for the excitatory event. The action spectrum of the achromatic response of the epiphysis and the frontal organ peaked between 500 and 520 nm and showed no Purkinje-shift during dark adaptation (Korf et al. 1981). The action spectrum of the early receptor potential of the anuran pineal complex closely resembled the absorption of rhodopsin indicating that the true pineal photoreceptors whose structure resembles cones contain a photopigment different from the retinal cones of the frog’s lateral eye (Morita and Dodt 1975). In line with these data, most true pineal photoreceptors bind antibodies against bovine rod-opsin (Vigh-Teichmann and Vigh 1990; Vigh-Teichmann et al. 1982; 1983) (Fig. 5) (for more details on photopigments, see Ekström and Meissl 1997; Meissl and Ekström 1993; Solessio and Engbretson 1993). Notably, modified pineal photoreceptors of the quail were also shown to display opsin immunoreaction (Foster et al. 1987) (Fig. 6). This conforms to the study by T. Deguchi (1981) showing a photopigment with an absorption maximum of rhodopsin. Even mammalian pinealocytes express proteins which are specific to retinal and pineal photoreceptors, such as rod-opsin (Korf et al. 1985a) or the S-antigen (rod arrestin) (Korf et al. 1985b) (Fig. 6) supporting the concept that they belong to a common cell lineage with true pineal photoreceptors. However, the immunoreactions in mammalian pinealocytes do not indicate their direct photosensitivity, since mammalian pineal organs in the adult state lack retinal derivatives as essential components of all known vertebrate photopigments (Kramm et al. 1993). Pinealocytes of the receptor line also comprise neuronal markers, such as neurofilaments or synaptophysin (Fig. 6) (Huang et al. 1992).

In addition to rod-opsin, several other opsins have been identified in true and modified pineal photoreceptors, such as pinopsin (Okano et al. 1994), parapinopsin (Blackshaw and Snyder 1997; Kawano-Yamashita et al. 2007), and melanopsin (Chaurasia et al. 2005).

**Fig. 7:**
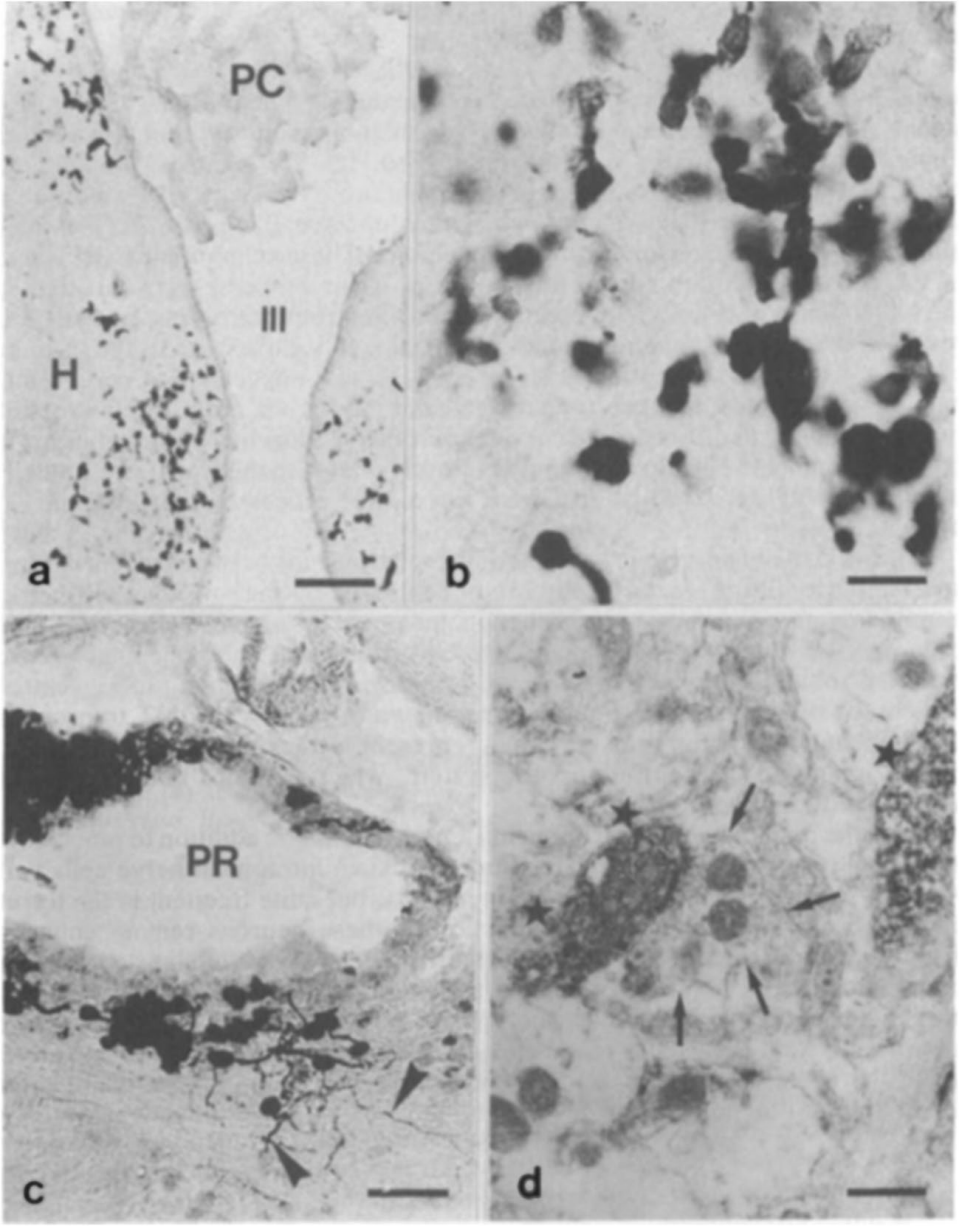
Interrelationship between the pineal organ and the epithalamic region (habenular nucleus, pretectal area) as shown by S-antigen immunocytochemistry. **a**, **b** Several cells in the rat medial habenular nucleus display S-antigen immunoreaction and are considered “displaced” pinealocytes (Rodriguez et al. 1988). H, habenular nucleus; III, third ventricle; PC, plexus choroideus. Bar = 100 µm (**a**) and 20 µm (**b**). **c** S-antigen immunoreactive pinealocytes in the proximal portion of the mouse pineal organ give rise to long axonlike processes (arrowheads) penetrating into the brain (Korf et al. 1990). PR, pineal recess. Bar = 40 µm. **d** Electron microscopic demonstration of S-antigen immunoreactive pinealocyte processes (stars) in the habenular nucleus of the mouse. Preembedding method. An immunonegative terminal containing clear synaptic vesicles (arrows) forms a conventional synapse with one of the immunolabeled processes (Korf et al. 1990). Bar = 5 µm

True pineal photoreceptors of poikilothermic vertebrates are synaptically connected to the intrapineal second-order neurons which give rise to the pineal tract targeting several areas in the diencephalon and mesencephalon. The intrapineal neurons were best visualized by means of the histochemical demonstration of acetylcholine esterase (Wake 1973; Wake et al. 1974; Korf 1974). These pinealofugal neuronal projections that convey neuronal signals to various brain centers are reduced in the course of phylogeny. Nevertheless, direct neuronlike connections appear to exist between the pineal organ and the central nervous system of mammals. These projections originate from a population of pinealocytes (Korf et al. 1986, 1990) (Fig. 7). Their functional significance remains an issue not yet resolved.

Irrespective of the phylogenetic transformation, the pineal organ of all vertebrates investigated thus far is capable of producing and releasing melatonin. Melatonin is rhythmically synthesized and released during darkness and, thus, represents an important neuroendocrine information on the ambient photoperiod (“hormone of darkness”). Melatonin levels mirror the length of the night and, thus, provide an essential signal for the control of seasonal rhythms in photoperiodic mammals (Hoffmann and Reiter 1965; Reiter 1991).

**Fig. 8:**
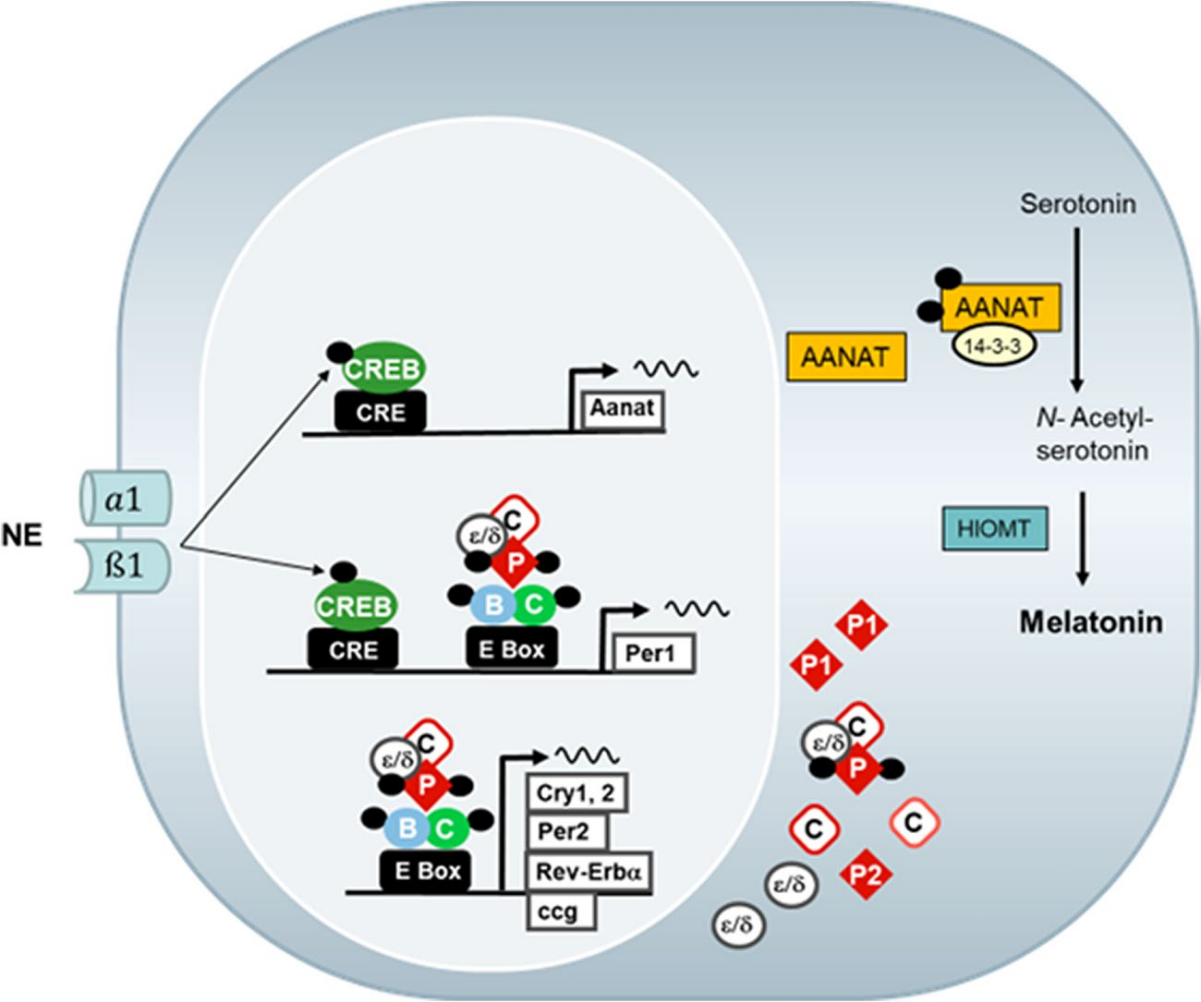
Norepinephrine-dependent signal transduction and gene expression in rodent pinealocytes. Norepinephrine (NE), released from postganglionic fibers, activates alpha1 and beta1 adrenergic receptors. Respective signal transduction cascades lead to the phosphorylation of the transcription factor cAMP/Ca.^2+^ responsive element binding protein (CREB). The binding of phosphorylated CREB to CREs in the respective promoter regions activates the expression of arylalkylamine N-acetyltransferase (Aanat) and Per1. AANAT is posttranslationally modified by binding 14–3-3 proteins and catalyzes the conversion of serotonin to N-acetylserotonin. This is further converted into melatonin by O-methyltransferation mediated by hydroxyindole O-methyltransferase (HIOMT). PER1 is an essential component of the negative regulator complex that drives rhythmic expression of clock genes and clock-controlled genes (ccg) (from Korf and von Gall 2024, with permission)

In all vertebrates, rhythmic melatonin production is driven by the penultimate enzyme of melatonin biosynthesis, the arylalkylamine-N-acetyltransferase (Klein and Weller 1970, Coon et al. 1995; Korf and von Gall 2006). In mammals, the rhythm in AANAT depends on the release of norepinephrine (NE) from the sympathetic postganglionic fibers originating in the superior cervical ganglion. NE is released from sympathetic nerve fibers exclusively at night, activates alpha-1 and beta-1 adrenergic receptors, and finally causes an increase in intracellular cAMP levels. In rodents, transcriptional activation of the *Aanat* gene is the initial step (Fig. 8). This involves activation of cAMP-dependent protein kinase A which leads to phosphorylation of the transcription factor cAMP/Ca^2+^ responsive element binding protein (CREB) (Tamotsu et al. 1995). The binding of phosphorylated CREB to CREs in the promoter regions activates the expression of arylalkylamine N-acetyltransferase (*Aanat*). AANAT is posttranslationally modified and catalyzes the conversion of serotonin to N-acetylserotonin. This is further converted into melatonin by O-methyltransferation mediated by hydroxyindole O-methyltransferase (HIOMT). In ungulates and primates, pinealocytes constantly synthesize AANAT protein from continually available *Aanat* mRNA. During the day — in the absence of NE stimulation —, the protein is immediately destroyed by proteasomal proteolysis. At nighttime, elevated cAMP levels cause phosphorylation of AANAT by protein kinase A. This post-translational modification leads to the interaction of phosphorylated AANAT with regulatory 14–3-3 proteins, which protect AANAT from degradation (Ganguly et al. 2001). Increases in AANAT protein are paralleled by increases in enzyme activity (see Schomerus and Korf 2005). In conclusion, a common neuroendocrine principle, the nocturnal rise in melatonin, is controlled by strikingly diverse regulatory mechanisms. This diversity has emerged in the course of evolution and reflects the high adaptive plasticity of the melatonin-generating pineal organ.

Pineal organs also express a full set of clock genes known to be indispensable for sustained rhythm generation. PER1 is an essential component that drives rhythmic expression of clock genes and clock-controlled genes (ccg) bearing E-box elements in their promoter. In the mammalian pineal, *Per1* expression can be induced by the NE/pCREB cascade which is fundamental for the control of pineal physiology in mammals. *Per1* mRNA and PER1 protein accumulation coincide with timecourses of many other cyclicAMP inducible genes including *Aanat* whose promoter contains an E-box and can thus be classified as a clock-controlled gene (Fig. 8).

Notably, melatonin synthesis varies significantly among individual pinealocytes as shown in the rat (Rath et al. 2016), and two types of pinealocytes could be distinguished: one type (called alpha-pinealocyte) efficiently O-methylates N-acetylserotonin that is produced and released by beta-pinealocytes, thereby improving the overall efficiency of melatonin synthesis (Mays et al. 2018). According to current knowledge, melatonin is not stored within pinealocytes but, upon formation, is immediately released into the bloodstream or the cerebrospinal fluid.

The rhythmic production of melatonin is under the control of circadian rhythm generators and photoreceptor cells. In several nonmammalian species, photoreceptors, circadian rhythm generators, and neuroendocrine effectors are colocalized in the pineal. Photoreceptive and neuroendocrine functions may be even located in one cell, which has been denominated as “photoneuroendocrine cell” by Andreas Oksche (Oksche 1984; Oksche and Hartwig 1979; Tamotsu et al. 1990). Later on, it was concluded that photoneuroendocrine cells also comprise a circadian rhythm generator (Korf 1994). The basis for adding a circadian rhythm generator to the definition of a “photoneuroendocrine cell” was laid by Takeo Deguchi showing that the isolated chicken pineal organ is capable of rhythm generation (Deguchi 1979).

In mammals, rhythmic melatonin synthesis is controlled by “extrapineal” signals transmitted from nonvisual photoreceptors located in the inner retina (see below) and the central rhythm generator in the suprachiasmatic nuclei (SCN) of the hypothalamus. Transmission of these signals involves a complex neuronal chain, whose last member is the sympathetic innervation originating from the superior cervical ganglion. As described above, this innervation is mandatory to maintain the rhythm of the melatonin biosynthesis in the mammalian pineal organ. Interestingly, norepinephrine, the major neurotransmitter in the sympathetic nerve fibers, elicits opposite effects on melatonin biosynthesis in birds and mammals: it stimulates the melatonin biosynthesis in the mammalian pineal organ but inhibits the melatonin formation in the chicken. This conversion occurs at the level of the adrenoreceptors.

### Pineal messengers other than melatonin

**Fig. 9:**
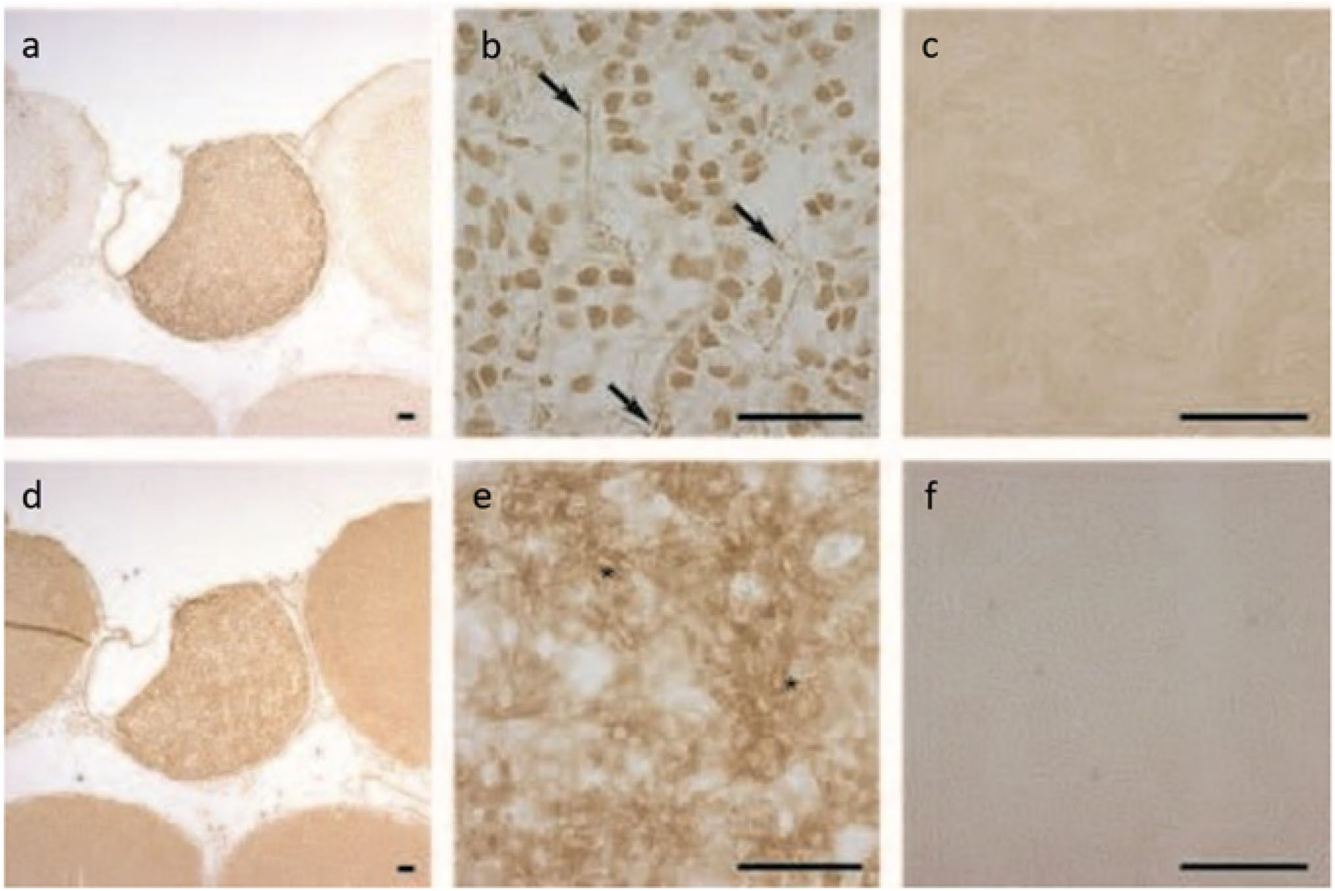
Immunohistochemical demonstration of two enzymes involved in endocannabinoid synthesis and degradation, N-acyl phosphatidyl ethanolamine hydrolyzing phospholipase D (NAPE-PLD), and fatty acid amide hydrolase (FAAH) in coronal brain sections obtained from male rats kept under 12 h light:12 h dark cycle with light on at zeitgeber time 0 (ZT00) and light off at ZT12. Scale bars = 50 µm. **a** NAPE-PLD immunoreaction was found in virtually all pinealocytes. **b** The NAPE-PLD immunoreaction was associated with the pinealocyte nuclei and was also found in varicosities and terminals of intrapineal nerve fibers running in the perivascular space (arrows). **c** Preabsorption of the NAPE-PLD antibody with the corresponding blocking peptide abolished the NAPE-PLD immunoreaction. **d** FAAH immunoreaction was evenly distributed in the pineal gland. **e** The FAAH signal was exclusively located in the cytoplasm of numerous pinealocytes (stars). **f** Preabsorption of the FAAH antibody with the corresponding blocking peptide abolished the FAAH immunoreaction. Reproduced from Koch et al. (2008) with permission

It has been repeatedly suggested that the pineal organ synthesizes and secretes other substances than melatonin. In the rat pineal gland, the endocannabinoid arachidonoyl ethanolamide (AEA) showed rhythmic changes with higher levels during the light period and reduced amounts at the onset of darkness. Norepinephrine, the essential stimulus for nocturnal melatonin biosynthesis, acutely down-regulated AEA and other endocannabinoids in cultured pineal glands. These temporal dynamics suggest that AEA exerts time-dependent autocrine and/or paracrine functions within the pineal. Moreover, endocannabinoids may be released from the pineal into the CSF or bloodstream (Koch et al. 2015). Immunohistochemical and immunoblot analyses demonstrated CB1 and CB2 receptor proteins, N-acyl phosphatidyl ethanolamine hydrolyzing phospholipase D (NAPE-PLD), an enzyme catalyzing endocannabinoid biosynthesis as well as fatty acid amide hydrolase (FAAH), an endocannabinoid catabolizing enzyme, in pinealocytes and in pineal sympathetic nerve fibers identified by double immunofluorescence with an antibody against tyrosine hydroxylase (Fig. 9). Thus, the rat pineal gland comprises a full endocannabinoid system (Koch et al. 2008) indicating that endocannabinoids may be involved in the control of pineal physiology since cannabinoids attenuate norepinephrine-induced melatonin biosynthesis in the rat pineal gland by reducing arylalkylamine N-acetyltransferase activity without involvement of cannabinoid receptors (Koch et al. 2006). This effect was specific since cannabinoids did not influence the activity of hydroxyindole-O-methyltransferase (HIOMT), the last enzyme in melatonin biosynthesis.

Other substances produced by the pineal organ may be of peptidergic nature. Antibodies directed against secretory glycoproteins of the subcommissural organ (AFRU, ASO 470) were shown to cross-react with cells in the pineal organ of lamprey larvae, coho salmon, a toad, two species of lizards, domestic fowl, albino rat, and bovine. The AFRU-immunoreactive cells were identified as pinealocytes of the receptor line (pineal photoreceptors, modified photoreceptors, or classical pinealocytes, respectively). These findings support the concept that several types of pinealocytes exist, which differ in their molecular, biochemical, and functional features. They also indicate the possibility that the AFRU- and ASO-immunoreactive material found in certain pinealocytes might represent a proteinaceous or peptide compound, which is synthesized and released from a specialized type of pinealocyte in a hormone-like fashion (Rodriguez et al. 1988).

**Fig. 10:**
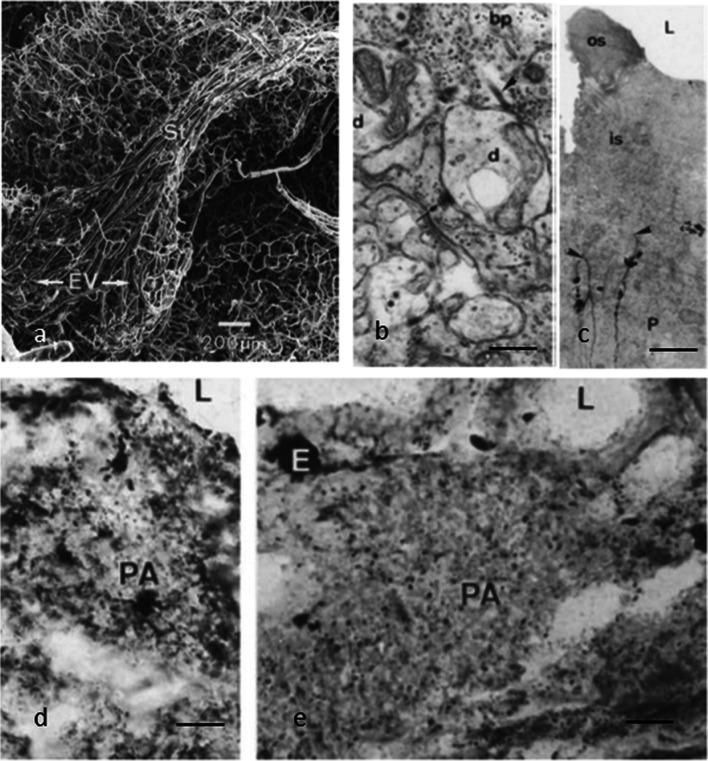
**a** Corrosion cast preparation of the vascular supply of the rainbow trout pineal organ. EV pineal end vesicle, St pineal stalk. Bar = 200 µm (Ali et al. 1987). **b**–**e** Blood–brain barrier is open in the rainbow trout pineal organ (Omura et al. 1985). **b** Neuropil in pineal parenchyma. Ferritin particles are confined to clear vesicles in receptor cells. Note that a synaptic ribbon (arrowhead) and a dense-core vesicle (asterisk) are free of the particles. Arrows indicate sensory synapses between basal processes (bp) of receptor cells and dendrites (d) of nerve cells. Twenty-four hours after intraperitoneal ferritin injection. **c** Intercellular passage of tannic acid is stopped at the junctional complex (arrowheads). Pineal lumen (L) free of the tracer. OS outer segment, IS inner segment of pineal photoreceptor (P). Bar = 5 µm. **d**, **e** Accumulation of horseradish peroxidase 24 h after intraperitoneal injection of the tracer. Pineal parenchyma (PA) displays many tracer granules, pineal lumen (L), erythrocytes (E). Bar in d 40 µm, in e 20 µm

Release of putative peptidergic messengers from the pineal would require an open blood–brain barrier and indeed several studies have revealed that the pineal organ lacks a blood–brain barrier as is typical for most of the circumventricular organs (rainbow trout, Omura et al. (1985); mouse, Møller et al. (1978); rat, Hewing and Bergmann (1985); gerbil, Hewing and Bergmann (1985); hamster, Hewing and Bergmann (1985); Chen et al. (1994)) (Fig. 10).

Ontogenetic studies have shown a differential maturation of retinal and pineal photoreceptors during development. In the clawed toad, *Xenopus laevis*, correlations between the ontogenetic occurrence of two photoreceptor-specific proteins, S-antigen and rod-opsin, and the maturation of the neurohormonal effector system involved in melatonin-dependent color-change mechanisms showed that (i) the molecular mechanisms of photoreception develop simultaneously in retina and pineal complex; (ii) most pineal photoreceptors differ from retinal rods in that they contain immunoreactive S-antigen but essentially no immunoreactive rod-opsin; and (iii) the differentiation of phototransduction processes coincides with the onset of melatonin-dependent photoneuroendocrine regulation of color-change mechanisms (B. Korf et al. 1989). On the other hand, the pineal complex of teleost fish matures earlier than the retina and has the ability to perceive light information much earlier than the retina (van Veen et al. 1984, Östholm et al. 1987). In general, nonvisual photoreceptors appear to develop earlier in phylogeny and ontogeny than visual photoreceptors. This holds also true for the nonvisual photoreceptors in the retina of the lateral eyes (Berson et al. 2002; Contin et al. 2006; Do and Yau 2010).

## Nonvisual photoreceptors in the inner retina of the lateral eye

**Fig. 11:**
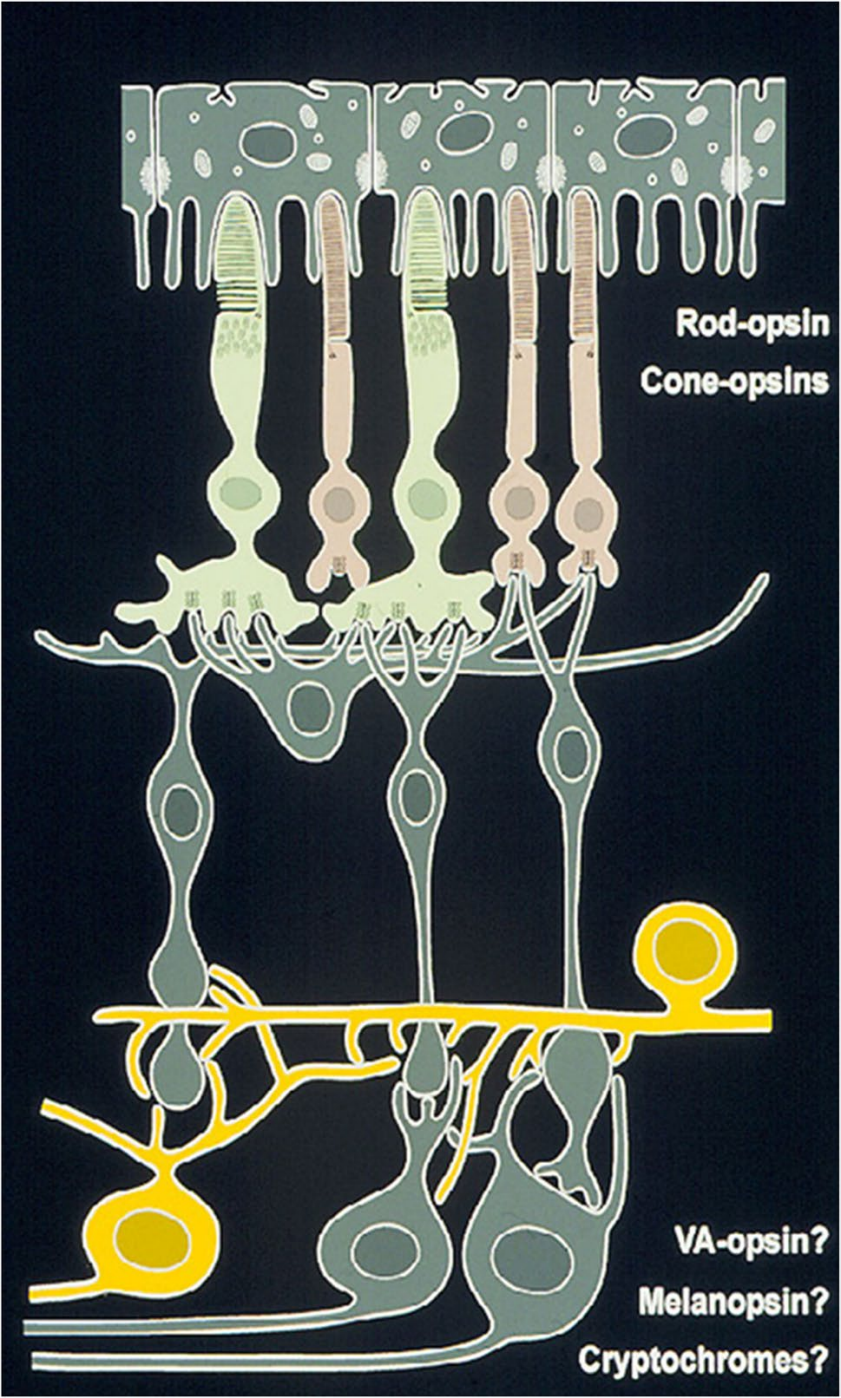
The retina comprises visual photoreceptors (rods, cones) with their specific opsins (rod-opsin, cone opsins) in the outer retinal layer and intrinsically photoreceptive ganglion cells and amacrine cells in the inner retinal layer. The latter serve as luminance detectors and contain melanopsin (Hattar et al. 2002) or other photopigments (cryptochromes, vertebrate ancient opsin). In mammals, the intrinsically photoreceptive ganglion cells employ the neuropeptide PACAP as a transmitter and their axons form the retinohypothalamic tract. In addition, they are capable of melatonin synthesis

In mammals, which, according to current knowledge, lack encephalic and pineal photoreceptors (at least in the postnatal stage), the retina harbors nonvisual photoreceptors, which are located in the ganglion cell layer, lack specialized outer segments, and contain a peculiar photopigment, denominated as melanopsin (Opn4) (Fig. 11). Melanopsin was discovered in directly photosensitive melanophores of the tailfin of *Xenopus laevis* larvae by Mark Rollag and colleagues (Provencio et al. 1998) and later on found to be conserved in the course of evolution (Provencio et al. 2000, Brainard et al. 2001). Melanopsin is retinaldehyde-based; its peak absorbance is in the blue region of the spectrum and distinct from that of rod and cone cell photopigments for vision. The human melanopsin gene consists of ten exons and is mapped to chromosome 10q22. These chromosomal localization and gene structure differ significantly from that of other human opsins that typically have four to seven exons. The deduced amino acid sequence of melanopsin shares greatest homology with cephalopod opsins. The predicted secondary structure of melanopsin indicates the presence of a long cytoplasmic tail with multiple putative phosphorylation sites, suggesting that its function may be finely regulated. A survey of 26 anatomical sites indicates that, in humans, melanopsin is expressed only in the eye. In situ hybridization histochemistry shows that melanopsin expression is restricted to cells within the ganglion and amacrine cell layers of the primate and murine retinae (Provencio et al. 2000). In the chicken retina, melanopsin was expressed in ganglion and horizontal cells (Contin et al. 2006; Morera et al. 2016). Notably, melanopsin expression is not observed in the photoreceptor layer of the outer retina where rods and cones are located that initiate vision. The unique inner retinal localization of melanopsin suggests that it is not involved in image formation but rather mediates nonvisual photoreceptive tasks, such as the entrainment of circadian rhythms and the acute suppression of pineal melatonin. The anatomical distribution of melanopsin-positive retinal cells is similar to the pattern of cells that form the retinohypothalamic tract, a projection from the retina to the suprachiasmatic nuclei of the hypothalamus, the primary circadian rhythm generator in mammals (Hannibal et al. 2014).

Melanopsin photoreceptive cells cooperate with classical rods and cones. Transgenic mice lacking melanopsin still retain nonvisual photoreception, suggesting that rods and cones could operate in this capacity. Mice with both outer-retinal degeneration and a deficiency in melanopsin exhibited complete loss of photoentrainment of the circadian rhythm generator, pupillary light responses, photic suppression of pineal melatonin biosynthesis (arylalkylamine-N-acetyltransferase transcript), and acute suppression of locomotor activity by light. This indicates the importance of both nonvisual and classical visual photoreceptor systems for nonvisual photic responses in mammals (Panda et al. 2003).

Like the pineal organ, the retina is capable of synthesizing melatonin via the same biosynthetic pathway that also operates in the pineal (Tosini and Menaker 1996; Tosini et al. 2012; Iuvone et al. 2005). However, the amount of melatonin produced by the retina is small compared to that in the pineal gland, the primary source of circulating melatonin, and retinal melatonin is thought to act as a local neuromodulator within the eye. Melatonin is synthesized in classical photoreceptors (Cahill and Besharse 1993) as well as in melanopsin containing ganglion and horizontal cells (Garbarino-Pico et al. 2004; Morera et al. 2016). In most vertebrate species, retinal melatonin synthesis and levels are high during the night and low during the day (Tosini et al. 2008); however, retinal melatonin levels are high during the daytime in trout (Iigo et al. 1997), chicken (Contin et al. 2006), and a diurnal rodent (Gianesini et al. 2015). In the vast majority of the species investigated thus far, melatonin synthesis in the retina is under the control of retinal circadian clocks since the retinae of fish, amphibians, reptiles, birds, and mammals synthesize melatonin in the rhythmic fashion when they are maintained in vitro under constant darkness (reviewed in Iuvone et al. 2005). Expression of melanopsin, clock genes, and the key melatonin synthesizing enzyme, arylalkylamine N-acetyltransferase (AA-NAT), appears very early in development in both cell populations suggesting that nonvisual photoreceptors develop at early developmental stages (Diaz et al. 2014).

## Deep brain (encephalic) photoreceptors

As already suggested by the early experiments by von Frisch, a third set of photoreceptors outside the retina and the pineal complex seems to exist in non-mammalian vertebrates. It has been known for many decades that such encephalic photoreceptors are involved in the photoperiodic control of the seasonal cycle of reproduction. An action spectrum for this response described an opsin photopigment with a λmax of ∼ 492 nm (Foster et al. 1985), but the precise location of the deep encephalic photoreceptors as well as the the specific identity of the photopigment has remained enigmatic for a long time.

Considering the morphological correlate of extraretinal and extrapineal photoreception, it is of note that nonmammalian vertebrates are endowed with a widespread system of cerebrospinal fluid (CSF)-contacting neurons. This system was discovered and extensively described by Vigh and Vigh-Teichmann (cf. Vigh and Vigh-Teichmann 1998, for a comprehensive synopsis of the system; for a recent review, see Wyart et al. 2023). Neurons may contact the ventricular CSF via their dendrites, axons, or perikarya. Most of the CSF-contacting neurons send their dendritic processes into the ventricular cavity, where they form ciliated terminals. These ciliated endings resemble those of known sensory cells. By means of axons, the CSF-contacting neurons also may contact the external CSF space, where the axons form terminals of a neurohormonal type similar to those known in the neurohemal areas.

In the hypothalamus, a conspicuous accumulation of CSF-contacting neurons is found in the paraventricular organ (PVO). Here, the neurons form three layers, they contain serotonin immunoreactivity, and the outer layer of neurons is dopaminergic. These neurons send their axons into the median eminence where they are in close contact with gonadotropin-releasing hormone (GnRH) immunoreactive nerve fibers. In 2010, Nakane et al. (2010) succeeded in demonstrating Opsin 5 (OPN5; also known as Gpr136, Neuropsin, PGR12, and TMEM13) mRNA in the paraventricular organ (PVO) of the Japanese quail. Immunohistochemistry identified Opsin 5 in CSF-contacting neurons and their axons extending to the external zone of the median eminence adjacent to the pars tuberalis of the pituitary gland, which translates photoperiodic information into neuroendocrine responses. The action spectrum showed peak sensitivity (lambda(max)) at approximately 420 nm and short-wavelength light stimuli between UV-B and blue light-induced photoperiodic responses in eye-patched, pinealectomized quail. Thus, Opsin 5 appears to be one of the deep brain photopigments that regulate seasonal reproduction in birds.

**Fig. 12:**
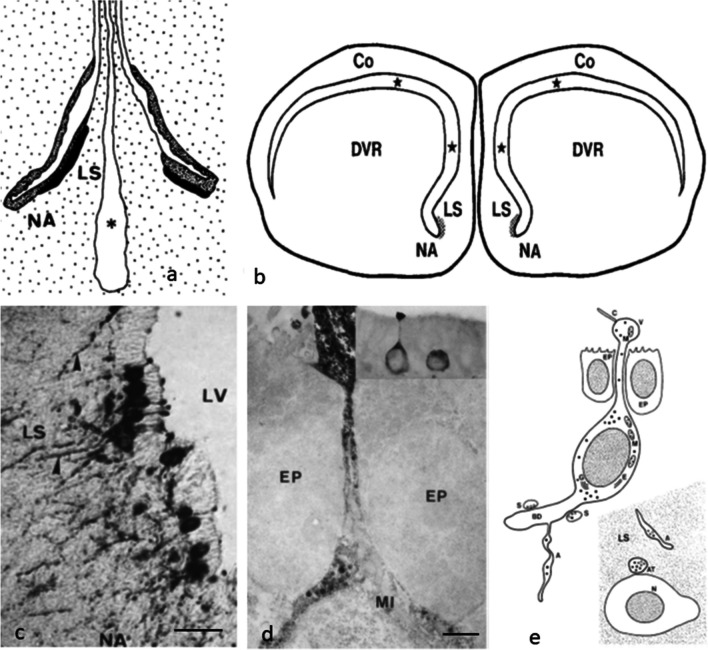
Location of the lateral septal organ. **a** Frontal section through the mallard brain (Korf and Fahrenkrug 1984). **b** Frontal section through the brain of the Nile crocodile. LS lateral septum, NA nucleus accumbens, DVR dorsoventral ridge, Co Cortex (Hirunagi et al. 1994). **c** Numerous VIP-immunoreactive CSF contacting neurons send their apical dendrites into the cerebrospinal fluid of the lateral ventricle (LV) where they show a bulbous swelling. Bar = 30 µm. **d** Immunoelectron microscopy of a VIP-immunoreactive CSF contacting neuron shown in the insert. The CSF contacting dendrite runs between two ependymal cells (EP) and terminates with a bulbous swelling. Bar = 5 µm. **e** Diagrammatic representation of direct contacts between VIP immunoreactive CSF-contacting neurons of the LSO and a GnRH immunoreactive neuron in the lateral septum. BD basal dendrite, A axon, AT VIP immunoreactive axon terminal contacting a GnRH immunoreactive neuron in the lateral septum (Kiyoshi et al. 1998)

A prominent CSF-contacting neuronal area was also found in the telencephalon in the region of the nucleus accumbens and the lateral septum (Korf and Fahrenkrug 1984) which has been denominated as lateral septal organ (Kuenzel and van Tienhoven 1982). This aggregation of CSF-contacting neurons was immunolabeled with antibodies against chicken vasoactive intestinal polypeptide (VIP). Deeper layers of the lateral septum and the nucleus accumbens are richly innervated by VIP-immunoreactive nerve fibers (Fig. 12).

As shown in pigeon and mallard, VIP-like immunoreactive cerebrospinal fluid (CSF)-contacting neurons project to small, presumably peptidergic nerve cells of the lateral septum suggesting that VIP serves as a neuromodulator (-transmitter) in this area (Hirunagi et al. 1994, 1995). By use of immunohistochemical single- and double-labeling techniques and electron microscopy, synaptic contacts between VIP-like-ir axon terminals and GnRH-like-ir cell bodies or dendrites were demonstrated suggesting functional interactions between VIP and GnRH neurons in the lateral septal-preoptic area (Kiyoshi et al. 1998) (Fig. 12). The lateral septal organ seems well conserved across species, it was also demonstrated in 11 reptilian (chelonian, lacertilian, ophidian, crocodilian) species (Hirunagi et al. 1993).

**Fig. 13:**
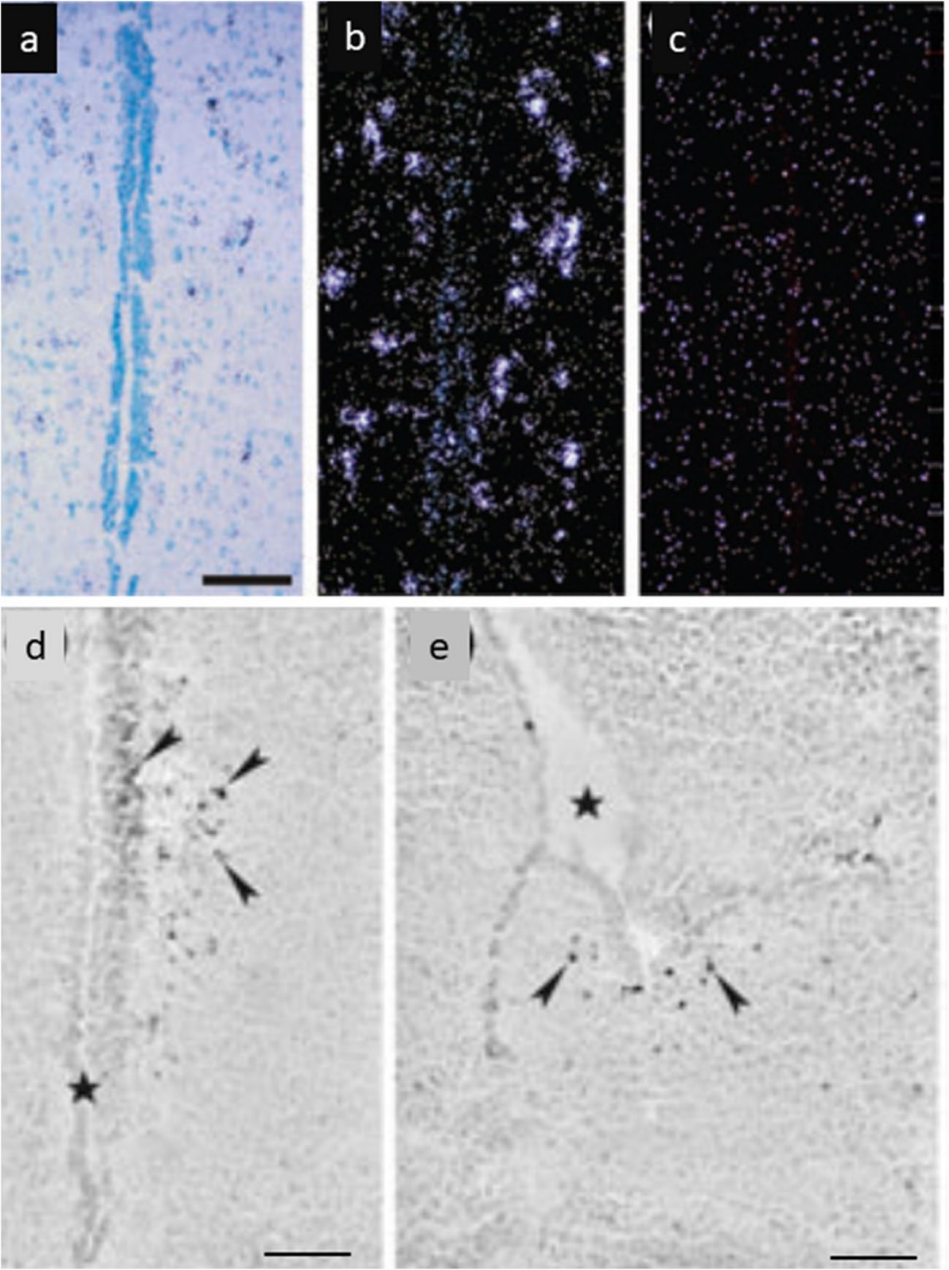
Melanopsin in situ hybridization histochemistry of deep brain photoreceptors in the chicken lateral septal organ. **a** Brightfield photomicrograph of a methylene green stained section of the chicken septum. **b** Darkfield photomicrograph of the same section after in situ hybridization of a melanopsin-specific riboprobe. **c** Darkfield photomicrograph of adjacent section hybridized with a sense control riboprobe. Bar = 100 µm. **d** Melanopsin expression in the lateral septal organ, rostral portion, and **e** caudal portion of the lateral septal organ of 1-day-old chicken. In situ hybridization with an anti-melanopsin riboprobe. Coronal frozen sections are slightly counterstained with cresyl violet. Arrowheads, selected labeled neurons. Bar = 100 µm. Reproduced from Chaurasia et al. (2005) with permission

With regard to the photopigments, several candidates have emerged including rod-opsin; melanopsin (OPN4); neuropsin (OPN5); and vertebrate ancient (VA) opsin. cDNA cloning of chicken melanopsin shows its expression not only in the retina and pineal organ but also in the brain where expression was observed in the lateral septal area (Fig. 13) and medial preoptic nucleus (Chaurasia et al. 2005). In the ring dove, CSF-contacting (CSF) neurons in both the septal and the tuberal areas are labeled by RET-P1, a monoclonal antibody to opsin that reacts with inner and outer segment membranes of rod photoreceptors in a variety of vertebrates. Double-label techniques demonstrated that RET-P1-positive cells of the lateral septal organ coexpress VIP-like immunoreactivity. VIP-positive cells in other brain areas are not RET-P1-positive (Silver et al. 1988). Also in passerine birds, the lateral septum was found to express rod-opsin (Wang and Wingfield 2011), and notably, two novel groups of rodopsin-immunoreactive cells were identified in the magnocellular part of paraventricular nucleus (PVN) of the hypothalamus and in the medial basal hypothalamus (MBH) of the white-crowned sparrow, *Zonotrichia leucophrys gambelii* (Zhao et al. 2018).

Apparently, also vertebrate ancient (VA) opsins play a role as photopigments of encephalic photoreceptors. These photopigments were isolated in 1997 and thought to have a restricted taxonomic distribution, confined to agnatha and teleost fish, but the isolation of VA opsin from chicken revealed that the two isoforms spliced from this gene (cVAL and cVA) are capable of forming functional photopigments and strongly implicate a role for VA opsin in mediating the avian photoperiodic response. Some studies suggest that VA opsin is co-expressed with both gonadotropin-releasing hormone (GnRH) and arginine-vasotocin (AVT) neurons and speculate that GnRH and AVT neurosecretory pathways are endogenously photosensitive (Garcia-Fernandez et al. 2015). Investigations on the role of VA and OPN5 in the avian photoperiodic response of Japanese quail, *Coturnix japonica*, suggested that the photoperiodic response involves at least two photoreceptor types and populations working together with VA opsin playing a dominant role (Perez et al. 2023).

In conclusion, the location of these encephalic photoreceptors seems to follow a general morphologic pattern (Bauplan): they are not randomly distributed but concentrated in two prime locations with CSF-contacting neurons, the lateral septal organ and the paraventricular organ. Notably, these photoreceptors are directly connected to the gonadotropin-releasing hormone (GnRH) system in the lateral septum and the median eminence (Kiyoshi et al. 1998, Saldanha et al. 2001; Nakane et al. 2010). Deep brain photoreceptors employ a variety of photopigments and occur in different brain regions. Opsins with absorption maxima in the blue/UV region, such as Opn2 (rhodopsin), seem to be expressed throughout the year while expression of Opn3 (encephalopsin) and Opn5 (neuropsin) displayed a seasonal rhythm (Marchese et al. 2024). Also, medaka was shown to undergo seasonal behavioral change accompanied by altered expression of opsin genes, resulting in reduced visual sensitivity to mates during winter-like conditions (Shimmura et al. 2017).

## The hypophysial pars tuberalis—an interface controlling seasonal functions

The pars tuberalis (PT) is an important interface between neuroendocrine centers in the hypothalamus and the pars distalis of the pituitary. The PT plays an essential role in the regulation of seasonal functions, in particular seasonal reproduction, and may even be the seat of the circannual clock (Lincoln et al. 2006). In nonmammalian vertebrates, light information on the season is perceived by encephalic photoreceptors such as the paraventricular organ. In mammals, the main input signal to the PT driving its physiology is melatonin. The duration of the melatonin signal decodes the ambient photoperiod: It is long in long nights and becomes shorter in spring when the nights get shorter (Reiter 1991).

**Fig. 14:**
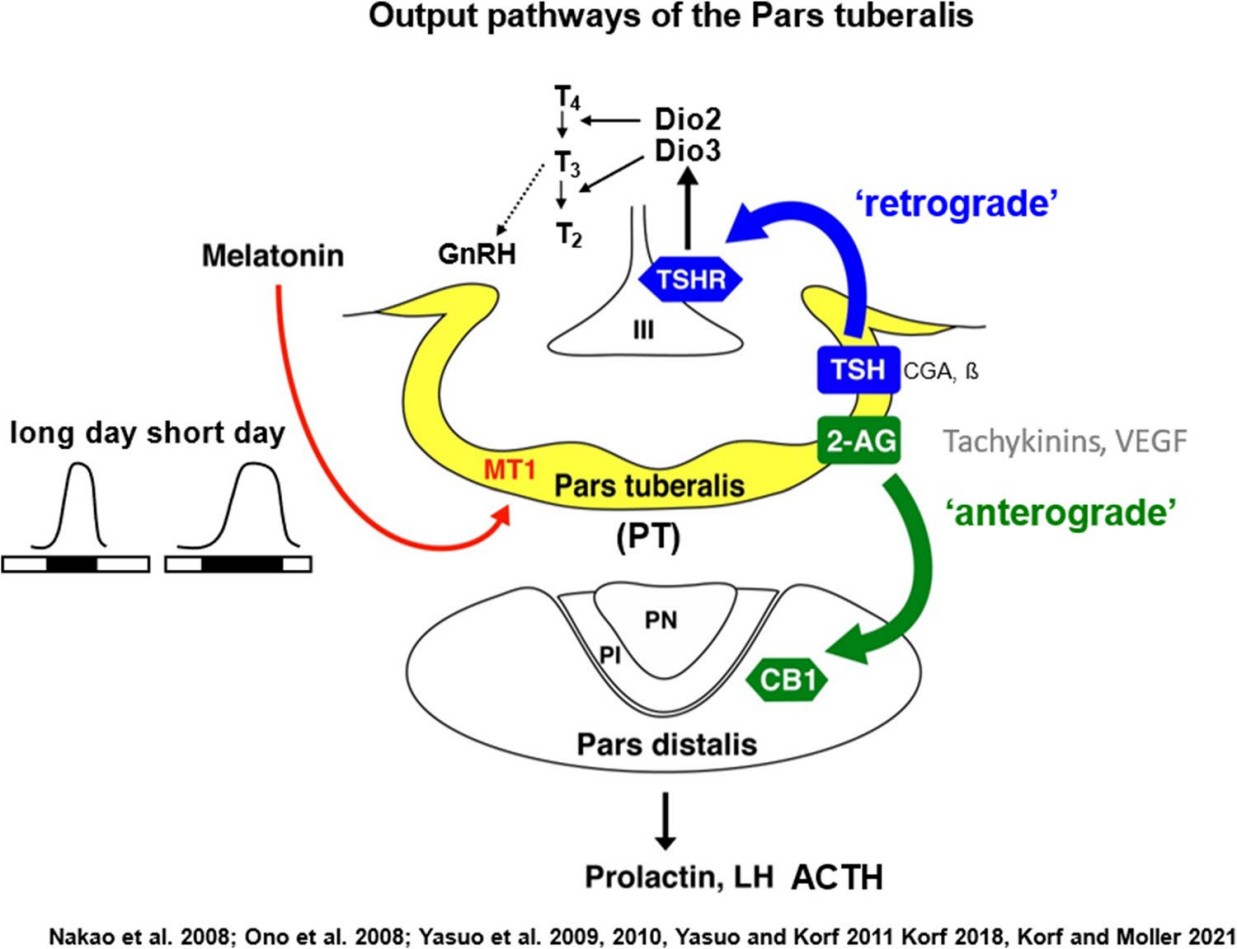
Schematic drawing of rodent pars tuberalis (PT) in the coronal plane and its input and output pathways. Photoperiodic information is transformed into melatonin signals acting on MT1 melatonin receptors expressed in the PT in high density, and various peptidergic and lipidergic messenger molecules are produced in the mammalian PT in response to the melatonin signal. Thyrotropin (TSH) comprises a common subunit and a specific beta subunit (TSHB). TSHB is a messenger molecule in the retrograde pathway from the PT to the ependymal cell layer of the third ventricle (tanycytes), which expresses the TSH receptor (TSHR) as well as type 2 (Dio2) and type 3 deiodinases. These enzymes regulate the local concentration of triiodothyronine (T3) that subsequently affects gonadotropin-releasing hormone (GnRH) secretion from axon terminals in the median eminence. Candidate messengers of the anterograde pathway are 2-arachidonoylglycerol (2-AG), tachykinins, and different splice forms of VEGF and TAFA3. They act on endocrine cells and folliculo-stellate cells in the pars distalis (PD) and affect angiogenesis in the portal vessels (for details, see text). III: third ventricle; PN: pars nervosa; PI: pars intermedia; ACTH: adrenocorticotropic hormone, LH: luteinizing hormone hormone; LH: luteinizing hormone. Reproduced from Korf (2018) with permission

**Fig. 15:**
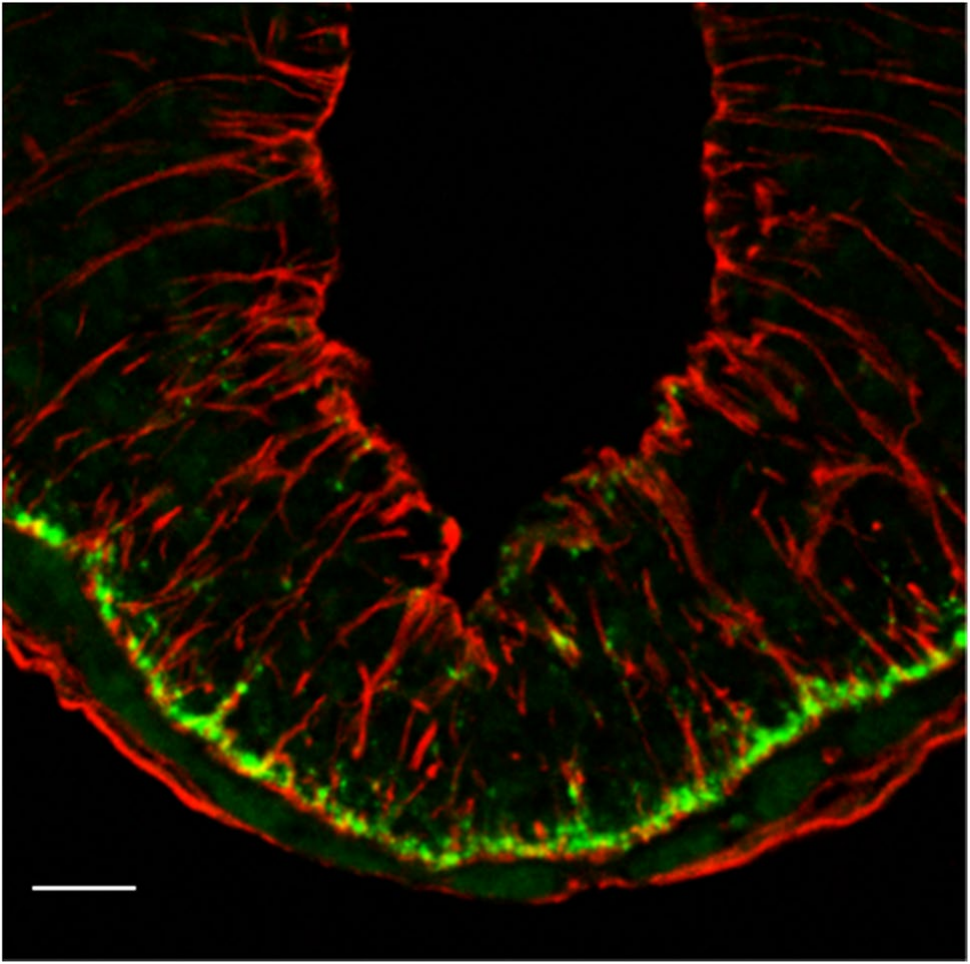
Median eminence of a melatonin-proficient C3H mouse. Endfeet of vimentin-immunoreactive tanycytes (red) engulf GnRH immunoreactive axons running in the outer layer of the median eminence (green). Bar = 50 µm

The PT transmits its signals via two output pathways: a retrograde pathway directed from the PT to the hypothalamus and an anterograde pathway directed from the PT to the hypophysial pars distalis (anterior lobe) (Fig. 14). The retrograde pathway of the PT employs thyroid stimulating hormone subunit beta (TSHB) as messenger and controls a local thyroid hormone system within the mediobasal hypothalamus via deiodinase (DIO) 2 and 3. This retrograde pathway has been discovered in Japanese quail (Yoshimura et al. 2003; Nakao et al. 2008). TSH triggers molecular cascades mediating thyroid hormone conversion in the ependymal cell layer of the infundibular recess of the third ventricle. The local accumulation of T3 in the mediobasal hypothalamus appears to activate the gonadal axis by stimulating the release of GnRH which involves neuro-glial interaction between GnRH terminals and tanycytes in the median eminence (Fig. 15). Tanycytes widely express the thyroidhormone receptor α (TRα) suggesting that the melatonin-driven T3 signal targets these nonneuronal cells (Quignon et al. 2020). Notably, PT-derived TSH does not affect thyroid functions, because it is rapidly degraded in the general circulation (Ikegami et al. 2014). The retrograde pathway is conserved in photoperiodic reproducing mammals (sheep and hamsters) and even in inbred mice provided that they are able to produce melatonin (Yasuo et al. 2009; Ono et al. 2008; Yasuo and Korf 2011; Korf 2018; Korf and von Gall 2024).

The anterograde pathway is implicated in the control of prolactin secretion, targets cells in the pars distalis, and employs small molecules as signal substances collectively denominated as “tuberalins.” Several “tuberalin” candidates have been proposed, such as tachykinins and endocannabinoids (EC). The PT-intrinsic EC system was first demonstrated in Syrian hamsters and shown to respond to photoperiodic changes (Yasuo et al. 2010a). Subsequently, the EC system was also demonstrated in the PT of mice, rats, and humans (Yasuo et al. 2010b). To date, 2-arachidonoylglycerol (2-AG) appears as the most important endocannabinoid from the PT. Likely, targets for the EC are folliculo-stellate cells of the pars distalis which contain the CB1 receptor and appear to contact lactotroph cells. The CB1 receptor was also found on corticotroph cells which appear as a further target of the EC. Taken together, the results support the concept that the PT transmits its signals via a “cocktail” of messenger molecules which operate also in other brain areas and systems rather than through PT-specific “tuberalins.” Furthermore, they may attribute a novel function to the PT, namely the modulation of the stress response and immune function known to display seasonal variation even in humans (Dopico et al. 2015).

## The discovery of clock genes

**Fig. 16:**
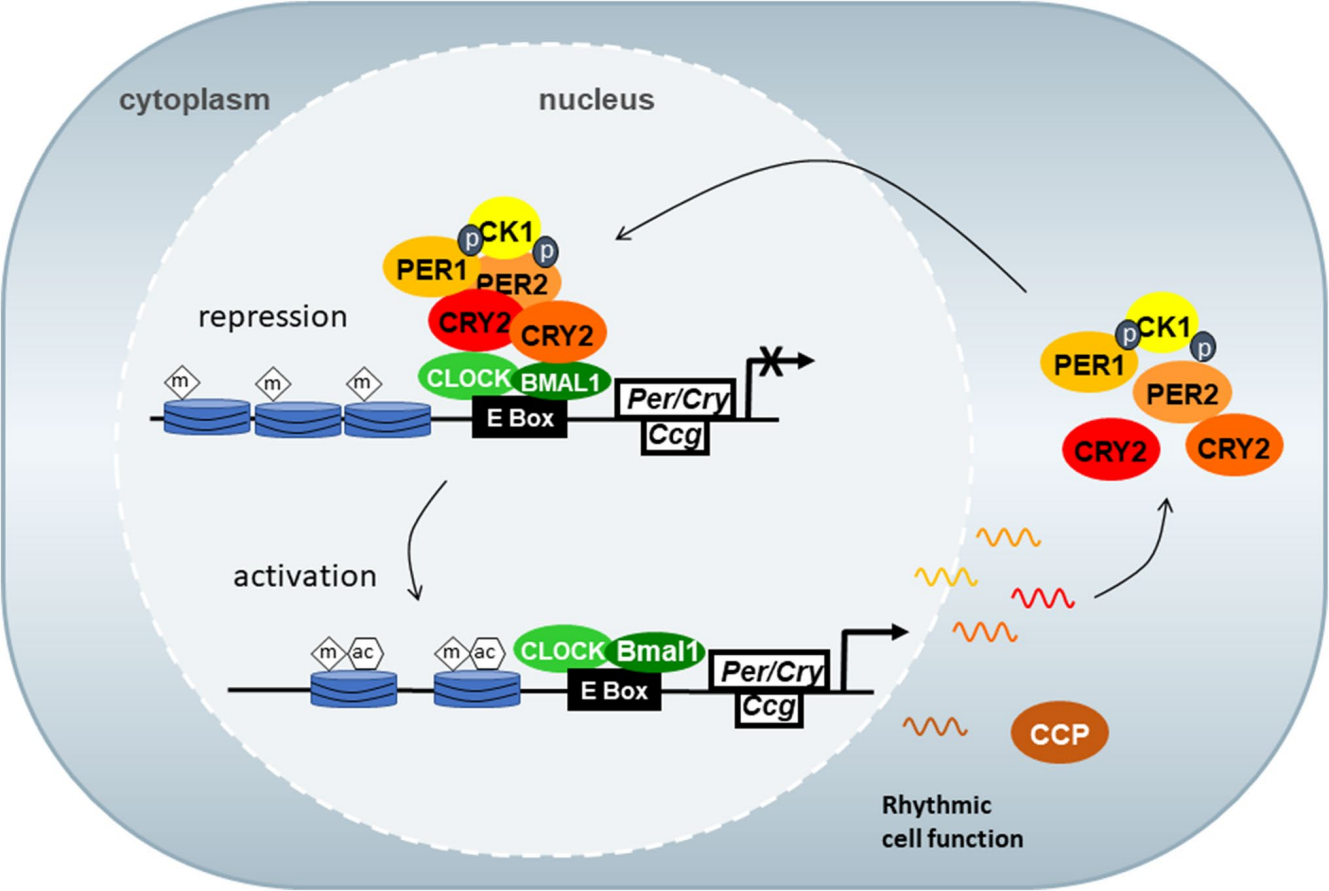
The molecular clockwork consists of transcriptional/translational feedback loops of clock genes. These encode transcription factors that activate (CLOCK and BMAL1) and inhibit (PER and CRY). BMAL1 (B) and CLOCK (C) activate the expression of Pers and Crys. PER and CRY proteins form a negative regulatory complex that in turn inhibits the activity of BMAL1 and CLOCK and thus their own expression. Post-translational modification, which is primarily mediated by casein kinases (CK1, ε/δ) and phosphatases, delays the breakdown of the negative regulatory complex, resulting in circadian periodicity of the feedback loop. Output of the molecular clockwork is provided through clock-controlled genes (Ccg) that encode clock-controlled proteins (CCP) which regulate the expression of 3000 genes involved in metabolism and cell proliferation. m methylation, ac acetylation. Reproduced from Korf and von Gall (2021) with permission

Research on biological rhythms was revolutionized after the discovery of clock genes in Drosophila (see Hall 2003; 2005; Luo et al. 2012; Abruzzi et al. 2011; Rosbash et al. 2007; Young 2018) for which Jeff Hall, Micheal Rosbash, and Michael Young were awarded with the Noble Prize for Medicine and Physiology in 2017. Later on, clock genes and molecular clockwork were identified in mammals (Fig. 16) (Sun et al. 1997; Albrecht et al. 1997; Antoch et al. 1997; King et al. 1997, Tei et al. 1997; Shigeyoshi et al. 1997; Yamaguchi et al. 2001; Shearman et al. 1997; Kume et al. 1999; see Korf and Stehle 2002, for review). These comparative studies clearly show that molecular clockwork is highly conserved during evolution and is of utmost importance for life and physiology.

**Fig. 17:**
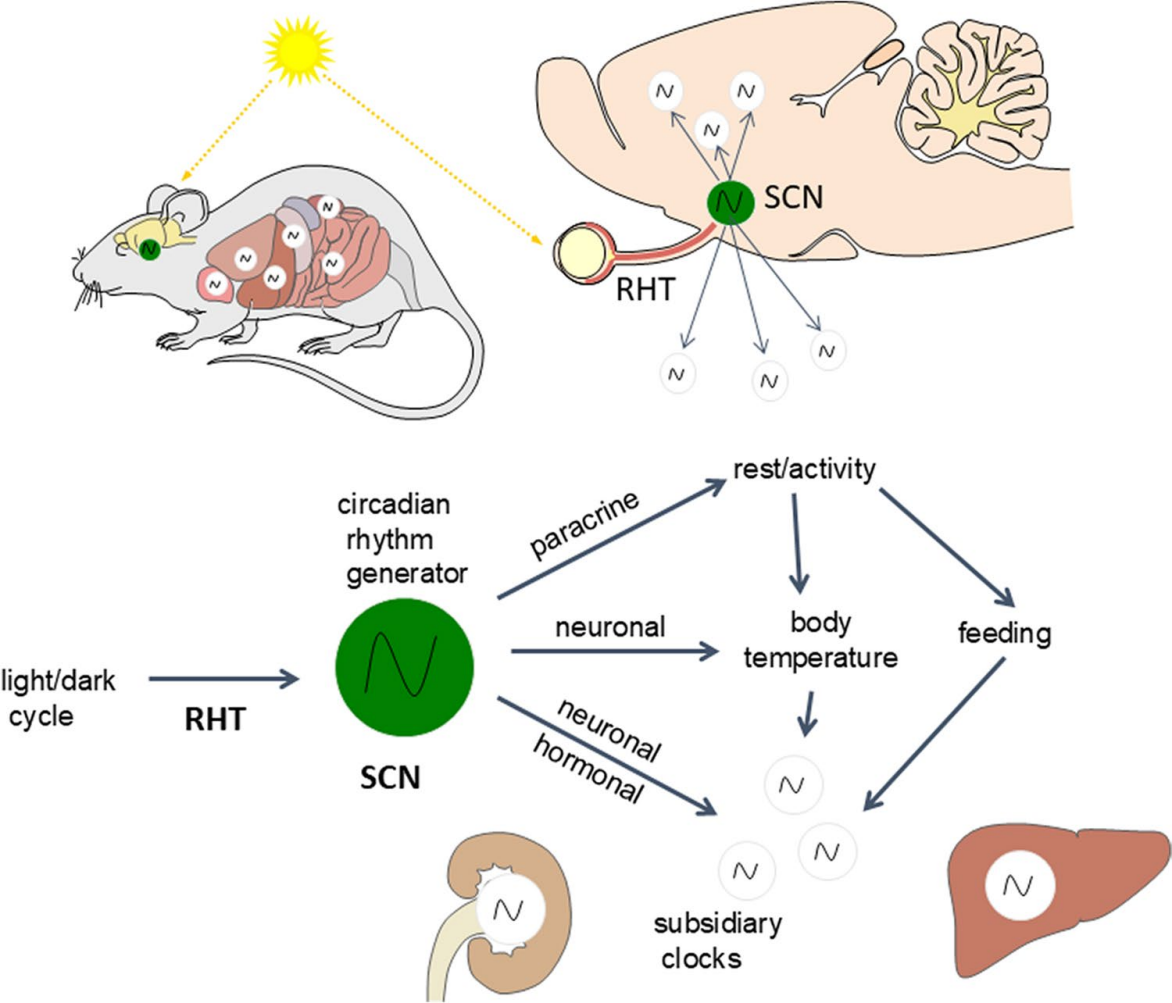
Hierarchy of the mammalian circadian system. The central rhythm generator is located in the bilateral suprachiasmatic nuclei of the hypothalamus (SCN). The SCN is entrained to the ambient photoperiod by light/dark information perceived in the retina by circadian photoreceptors employing melanopsin and transmitted to the SCN via the retinohypothalamic tract (RHT) which uses glutamate and PACAP as neurotransmitters. Output pathways of the SCN targeting peripheral oscillators are provided by paracrine mechanisms, neuronal pathways (sympathetic and parasympathetic nerve fibers), and hormones (glucocorticoids, melatonin). The system controls a variety of body functions, such as the sleep–wake cycle, body temperature, hormones, and metabolic activity (modified after Korf and von Gall 2021)

Molecular clockworks are ticking in each nucleated cell of the body, but in complex organisms such as mammals including man, the circadian system shows a remarkable, hierarchical organization: the central rhythm generator, e.g., the conductor of the system is located in the bilateral suprachiasmatic nuclei (SCN) of the hypothalamus which generate a self-sustained endogenous rhythm. The SCN orchestrates subordinate “peripheral” oscillators (e.g., liver, hippocampus, hypophysial pars tuberalis) via the autonomic nervous system (sympathetic and parasympathetic) (Buijs and Kalsbeek 2001) as well as glucocorticoids and melatonin as output signals (Fig. 17).

**Fig. 18:**
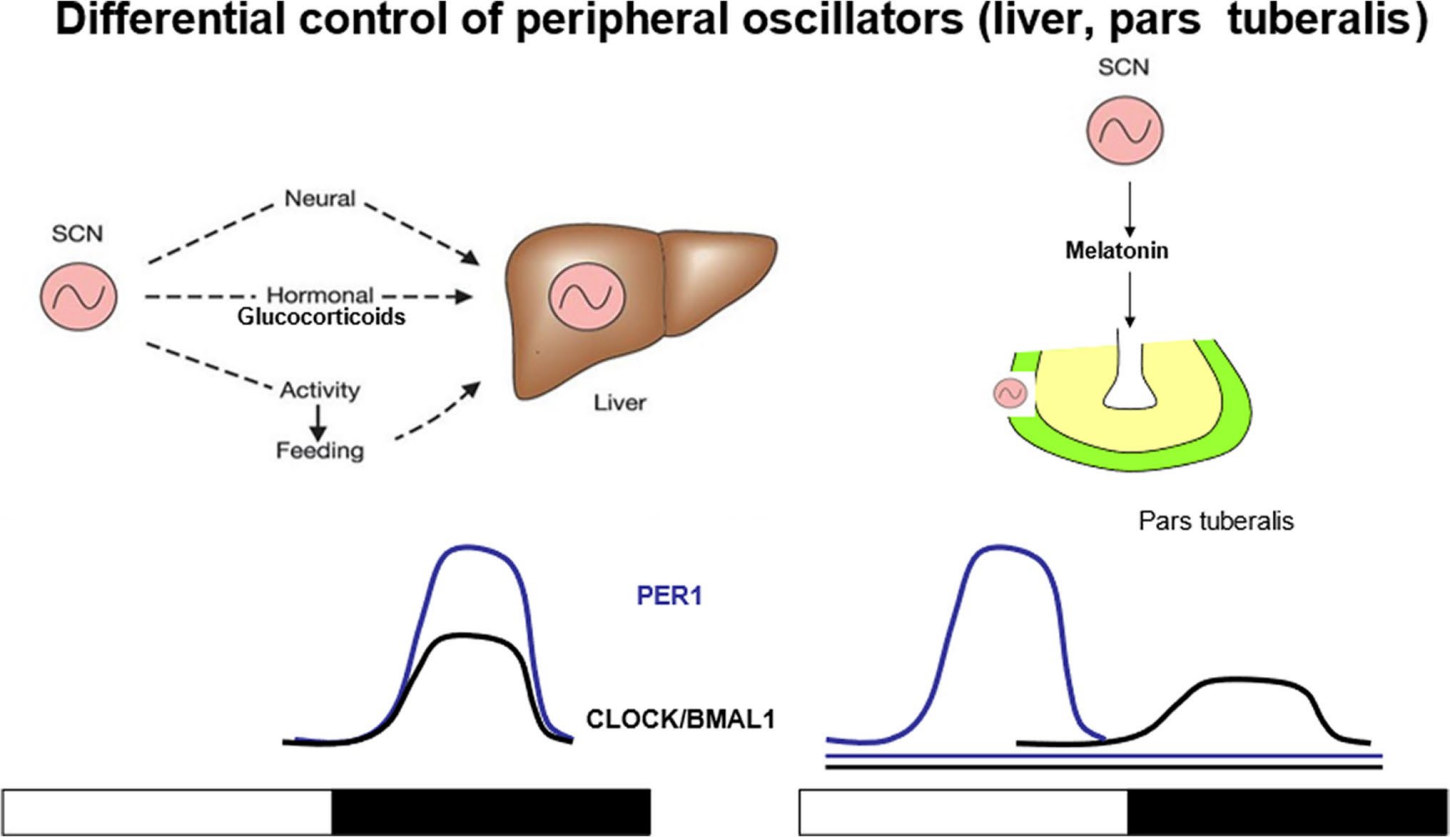
Synchronization of peripheral body clocks requires different agents. While the liver clock is synchronized by stimuli from the autonomic nervous system, glucocorticoid levels, and feeding (glucose concentration), synchronization of the pars tuberalis clockwork strictly depends on the melatonin signal

Melatonin, the hormone of darkness made in the pineal, is a prominent rhythmic hormonal output of the circadian system. It modulates the rhythmic activity of some, but not all subsidiary oscillators, and feeds back onto the SCN. Moreover, melatonin controls seasonal reproduction and metabolism in photoperiodic mammals and participates in the seasonal phase control of the cortisol rhythm (Chakir et al. 2015). While melatonin is essential to control the PT physiology, it is not needed to coordinate the liver. This organ strongly depends on glucocorticoids and feeding stimuli (Fig. 18).

## Future perspectives

**Fig. 19:**
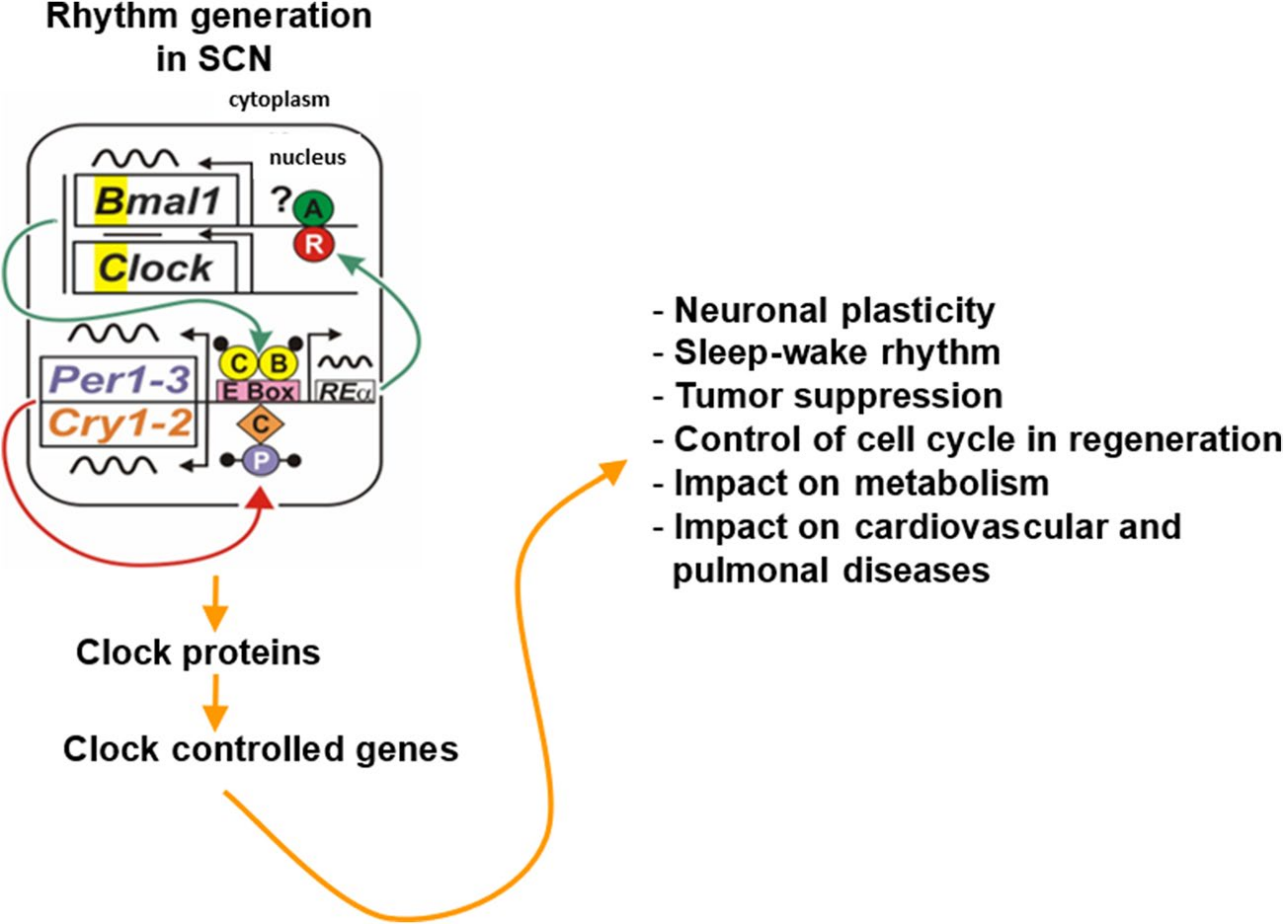
Body functions affected by the molecular clockwork and clock-controlled genes

When circadian rhythms are perturbed or misaligned, as a result of jet lag, shiftwork, or other lifestyle factors, adverse health consequences arise, and the risks of diseases such as cancer, cardiovascular diseases, or metabolic disorders increase (Fig. 19). Circadian disruption is associated with enhanced tumor formation and metastasis via dysregulation of key biological processes and modulation of cancer stem cells (CSCs) and their specialized microenvironment. Identifying circadian therapeutic targets could facilitate the development of new treatments that leverage circadian modulation to ablate tumor-resident CSCs, inhibit tumor metastasis, and enhance response to current therapies (Wang et al. 2024; Zheng et al. 2024). The timing of immunotherapy infusions affects survival and immunologic correlates in patients suffering from metastatic renal cell carcinoma (Patel et al. 2024). Chrono-tailored treatment strategies (Butler et al. 2024) will increase treatment efficacy and reduce adverse side effects advance (Hassan et al. 2021a, b). All these studies clearly show that time (timing) matters in medicine and it is now time to develop a new discipline: circadian medicine (Ruan et al. 2021; Kramer 2023).

## Data Availability

No datasets were generated or analysed during the current study.

## References

[CR1] Abruzzi KC, Rodriguez J, Menet JS, Desrochers J, Zadina A, Luo W, Tkachev S, Rosbash M (2011) Drosophila CLOCK target gene characterization: implications for circadian tissue-specific gene expression. Genes Dev 25(22):2374–2386. 10.1101/gad.178079.111 22085964 10.1101/gad.178079.111 PMC3222903

[CR2] Albrecht U, Sun ZS, Eichele G, Lee CC (1997) A differential response of two putative mammalian circadian regulators, mper1 and mper2, to light. Cell 91(7):1055–1064. 10.1016/s0092-8674(00)80495-x 9428527 10.1016/s0092-8674(00)80495-x

[CR3] Ali SS, Korf HW, Oksche A (1987) Microvasculature of the pineal organ of the rainbow trout (Salmo gairdneri). Cell Tissue Res 250(2):425–429. 10.1007/BF00219087 3427613 10.1007/BF00219087

[CR4] Antoch MP, Song EJ, Chang AM et al (1997) Functional identification of the mouse circadian Clock gene by transgenic BAC rescue. Cell 89(4):655–667. 10.1016/s0092-8674(00)80246-9 9160756 10.1016/s0092-8674(00)80246-9 PMC3764491

[CR5] Aschoff J, Hoffmann K, Pohl H, Wever R (1975) Re-entrainment of circadian rhythms after phase-shifts of the Zeitgeber. Chronobiologia. 2(1):23–78 1192905

[CR6] Berson DM, Dunn FA, Takao M (2002) Phototransduction by retinal ganglion cells that set the circadian clock. Science 295(5557):1070–1073. 10.1126/science.1067262 11834835 10.1126/science.1067262

[CR7] Blackshaw S, Snyder SH (1997) Parapinopsin, a novel catfish opsin localized to the parapineal organ, defines a new gene family. J Neurosci 17(21):8083–8092. 10.1523/JNEUROSCI.17-21-08083.1997 9334384 10.1523/JNEUROSCI.17-21-08083.1997 PMC6573767

[CR8] Brainard GC, Hanifin JP, Greeson JM, Byrne B, Glickman G, Gerner E, Rollag MD (2001) Action spectrum for melatonin regulation in humans: evidence for a novel circadian photoreceptor. J Neurosci 21(16):6405–6412. 10.1523/JNEUROSCI.21-16-06405.2001 11487664 10.1523/JNEUROSCI.21-16-06405.2001 PMC6763155

[CR9] Buijs RM, Kalsbeek A (2001) Hypothalamic integration of central and peripheral clocks. Nat Rev Neurosci 2(7):521–526. 10.1038/35081582 11433377 10.1038/35081582

[CR10] Butler CT, Rodgers AM, Curtis AM, Donnelly RF (2024) Chrono-tailored drug delivery systems: recent advances and future directions. Drug Deliv Transl Res 14:17556–1775. 10.1007/s13346-024-01539-4 10.1007/s13346-024-01539-4 PMC11153310 38416386

[CR11] Cahill GM, Besharse JC (1993) Circadian clock functions localized in xenopus retinal photoreceptors. Neuron 10(4):573–577. 10.1016/0896-6273(93)90160-s 10.1016/0896-6273(93)90160-s 8476609

[CR12] Chakir I, Dumont S, Pevet P, Ouarour A, Challet E, Vuillez P (2015) Pineal melatonin is a circadian time-giver for leptin rhythm in Syrian hamsters. Front Neurosci 9:190. 10.3389/fnins.2015.00190 26074760 10.3389/fnins.2015.00190 PMC4444759

[CR13] Chaurasia SS, Rollag MD, Jiang G, Hayes WP, Haque R, Natesan A, Zatz M, Tosini G, Liu C, Korf HW, Iuvone PM, Provencio I (2005) Molecular cloning, localization and circadian expression of chicken melanopsin (Opn4): differential regulation of expression in pineal and retinal cell types. J Neurochem 92(1):158–170. 10.1111/j.1471-4159.2004.02874.x 15606905 10.1111/j.1471-4159.2004.02874.x

[CR14] Chen YL, Chen WP, Huang BN, Lin HS (1994) Permeability of the pineal organ of the golden hamster (Mesocricetus auratus) to HRP with special reference to different types of blood capillaries. Arch Histol Cytol 57(2):175–186. 10.1679/aohc.57.175 8068407 10.1679/aohc.57.175

[CR15] Contin MA, Verra DM, Guido ME (2006) An invertebrate-like phototransduction cascade mediates light detection in the chicken retinal ganglion cells. FASEB J 20(14):2648–2650. 10.1096/fj.06-6133fje 17077288 10.1096/fj.06-6133fje

[CR16] Coon SL, Roseboom PH, Baler R, Weller JL, Namboodiri MA, Koonin EV, Klein DC (1995) Pineal serotonin N-acetyltransferase: expression cloning and molecular analysis. Science 270(5242):1681–1683. 10.1126/science.270.5242.1681 7502081 10.1126/science.270.5242.1681

[CR17] Dartnall HJ (1953) The interpretation of spectral sensitivity curves. Br Med Bull 9(1):24–30. 10.1093/oxfordjournals.bmb.a074302 10.1093/oxfordjournals.bmb.a074302 13032421

[CR18] Deguchi T (1979) A circadian oscillator in cultured cells of chicken pineal gland. Nature 282(5734):94–96. 10.1038/282094a0 503196 10.1038/282094a0

[CR19] Deguchi T (1981) Rhodopsin-like photosensitivity of isolated chicken pineal gland. Nature 290(5808):706–707. 10.1038/290706a0 7219554 10.1038/290706a0

[CR20] Díaz NM, Morera LP, Verra DM, Contin MA, Guido ME (2014) Early appearance of nonvisual and circadian markers in the developing inner retinal cells of chicken. Biomed Res Int 2014:646847. 10.1155/2014/646847 24977155 10.1155/2014/646847 PMC4055225

[CR21] Do MT, Yau KW (2010) Intrinsically photosensitive retinal ganglion cells. Physiol Rev 90(4):1547–1581. 10.1152/physrev.00013.2010 20959623 10.1152/physrev.00013.2010 PMC4374737

[CR22] Dodt E, Heerd E (1962) Mode of action of pineal nerve fibers in frogs. J Neurophysiol 25:405–429. 10.1152/jn.1962.25.3.405 13886872 10.1152/jn.1962.25.3.405

[CR23] Dopico XC, Evangelou M, Ferreira RC, Guo H, Pekalski ML, Smyth DJ, Cooper N, Burren OS, Fulford AJ, Hennig BJ, Prentice AM, Ziegler AG, Bonifacio E, Wallace C, Todd JA (2015) Widespread seasonal gene expression reveals annual differences in human immunity and physiology. Nat Commun 12(6):7000. 10.1038/ncomms8000 10.1038/ncomms8000 PMC4432600 25965853

[CR24] Ekström P, Meissl H (1997) The pineal organ of teleost fishes. Rev Fish Biol Fisheries 7:199–284

[CR25] Foster RG, Follett BK, Lythgoe JN (1985) Rhodopsin-like sensitivity of extra-retinal photoreceptors mediating the photoperiodic response in quail. Nature 313(5997):50–2. 10.1038/313050a0 3965970 10.1038/313050a0

[CR26] Foster RG, Korf HW, Schalken JJ (1987) Immunocytochemical markers revealing retinal and pineal but not hypothalamic photoreceptor systems in the Japanese quail. Cell Tissue Res 248(1):161–167. 10.1007/BF01239977 2952278 10.1007/BF01239977

[CR27] Ganguly S, Gastel JA, Weller JL, Schwartz C, Jaffe H, Namboodiri MA, Coon SL, Hickman AB, Rollag M, Obsil T, Beauverger P, Ferry G, Boutin JA, Klein DC (2001) Role of a pineal cAMP-operated arylalkylamine N-acetyltransferase/14-3-3-binding switch in melatonin synthesis. Proc Natl Acad Sci U S A 98(14):8083–8088. 10.1073/pnas.141118798 11427721 10.1073/pnas.141118798 PMC35471

[CR28] Garbarino-Pico E, Carpentieri AR, Contin MA, Sarmiento MI, Brocco MA, Panzetta P, Rosenstein RE, Caputto BL, Guido ME (2004) Retinal ganglion cells are autonomous circadian oscillators synthesizing N-acetylserotonin during the day. J Biol Chem 279(49):51172–51181. 10.1074/jbc.M309248200 15448149 10.1074/jbc.M309248200

[CR29] García-Fernández JM, Cernuda-Cernuda R, Davies WI, Rodgers J, Turton M, Peirson SN, Follett BK, Halford S, Hughes S, Hankins MW, Foster RG (2015) The hypothalamic photoreceptors regulating seasonal reproduction in birds: a prime role for VA opsin. Front Neuroendocrinol 37:13–28. 10.1016/j.yfrne.2014.11.001 25448788 10.1016/j.yfrne.2014.11.001

[CR30] Gianesini C, Clesse D, Tosini G, Hicks D, Laurent V (2015) Unique regulation of the melatonin synthetic pathway in the retina of diurnal female Arvicanthis ansorgei (Rodentia). Endocrinology 156(9):3292–3308. 10.1210/EN.2015-1267 26153723 10.1210/EN.2015-1267

[CR31] Guido ME, Marchese NA, Rios MN, Morera LP, Diaz NM, Garbarino-Pico E, Contin MA (2022) Non-visual opsins and novel photo-detectors in the vertebrate inner retina mediate light responses within the blue spectrum region. Cell Mol Neurobiol 42(1):59–83. 10.1007/s10571-020-00997-x 33231827 10.1007/s10571-020-00997-x PMC11441211

[CR32] Hafeez MA, Korf HW, Oksche A (1987) Immunocytochemical and electron-microscopic investigations of the pineal organ in adult agamid lizards. Uromastix Hardwicki Cell Tissue Res 250(3):571–578. 10.1007/BF00218948 3690636 10.1007/BF00218948

[CR33] Hall JC (2003) A neurogeneticist’s manifesto. J Neurogenet 17(1):1–90 14504029

[CR34] Hall JC (2005) Systems approaches to biological rhythms in Drosophila. Methods Enzymol 393:61–185. 10.1016/S0076-6879(05)93004-8 15817287 10.1016/S0076-6879(05)93004-8

[CR35] Hannibal J, Kankipati L, Strang CE, Peterson BB, Dacey D, Gamlin PD (2014) Central projections of intrinsically photosensitive retinal ganglion cells in the macaque monkey. J Comp Neurol 522(10):2231–2248. 10.1002/cne.23588 24752373 10.1002/cne.23588 PMC3996456

[CR36] Hassan SA, Ali AAH, Sohn D, Flögel U, Jänicke RU, Korf HW, von Gall C (2021) Does timing matter in radiotherapy of hepatocellular carcinoma? An experimental study in mice. Cancer Med 10(21):7712–7725. 10.1002/cam4.4277 34545699 10.1002/cam4.4277 PMC8559477

[CR37] Hassan SA, Ali AAH, Yassine M, Sohn D, Pfeffer M, Jänicke RU, Korf HW, von Gall C (2021) Relationship between locomotor activity rhythm and corticosterone levels during HCC development, progression, and treatment in a mouse model. J Pineal Res 70(3):e12724. 10.1111/jpi.12724 33615553 10.1111/jpi.12724

[CR38] Hattar S, Liao HW, Takao M, Berson DM, Yau KW (2002) Melanopsin-containing retinal ganglion cells: architecture, projections, and intrinsic photosensitivity. Science 295(5557):1065–1070. 10.1126/science.1069609 11834834 10.1126/science.1069609 PMC2885915

[CR39] Hewing M, Bergmann M (1985) Differential permeability of pineal capillaries to lanthanum ion in the rat (Rattus norvegicus), gerbil (Meriones unguiculatus) and golden hamster (Mesocricetus auratus). Cell Tissue Res 241(1):149–154. 10.1007/BF00214636 4028114 10.1007/BF00214636

[CR40] Hirunagi K, Rommel E, Oksche A, Korf HW (1993) Vasoactive intestinal peptide-immunoreactive cerebrospinal fluid-contacting neurons in the reptilian lateral septum/nucleus accumbens. Cell Tissue Res 274(1):79–90. 10.1007/BF00327988 8242714 10.1007/BF00327988

[CR41] Hirunagi K, Kiyoshi K, Adachi A, Hasegawa M, Ebihara S, Korf HW (1994) Electron-microscopic investigations of vasoactive intestinal peptide (VIP)-like immunoreactive terminal formations in the lateral septum of the pigeon. Cell Tissue Res 278(2):415–418. 10.1007/BF00414184 8001092 10.1007/BF00414184

[CR42] Hirunagi K, Rommel E, Korf HW (1995) Ultrastructure of cerebrospinal fluid-contacting neurons immunoreactive to vasoactive intestinal peptide and properties of the blood-brain barrier in the lateral septal organ of the duck. Cell Tissue Res 279(1):123–133. 10.1007/BF00300699 7895253 10.1007/BF00300699

[CR43] Hoffman RA, Reiter RJ (1965) Pineal gland: influence on gonads of male hamsters. Science 148(3677):1609–1611. 10.1126/science.148.3677.1609 14287606 10.1126/science.148.3677.1609

[CR44] Huang SK, Klein DC, Korf HW (1992) Immunocytochemical demonstration of rod-opsin, S-antigen, and neuron-specific proteins in the human pineal gland. Cell Tissue Res 267(3):493–498. 10.1007/BF00319371 1533347 10.1007/BF00319371

[CR45] Iigo M, Hara M, Ohtani-Kaneko R, Hirata K, Tabata M, Aida K (1997) Photic and circadian regulations of melatonin rhythms in fishes. Biol Signals. 6(4–6):225–32. 10.1159/000109132 9500660 10.1159/000109132

[CR46] Ikegami K, Liao XH, Hoshino Y et al (2014) Tissue-specific posttranslational modification allows functional targeting of thyrotropin. Cell Rep 9(3):801–810. 10.1016/j.celrep.2014.10.006 25437536 10.1016/j.celrep.2014.10.006 PMC4251493

[CR47] Iuvone PM, Tosini G, Pozdeyev N, Haque R, Klein DC, Chaurasia SS (2005) Circadian clocks, clock networks, arylalkylamine N-acetyltransferase, and melatonin in the retina. Prog Retin Eye Res 24(4):433–456. 10.1016/j.preteyeres.2005.01.003 15845344 10.1016/j.preteyeres.2005.01.003

[CR48] Kawano-Yamashita E, Terakita A, Koyanagi M, Shichida Y, Oishi T, Tamotsu S (2007) Immunohistochemical characterization of a parapinopsin-containing photoreceptor cell involved in the ultraviolet/green discrimination in the pineal organ of the river lamprey Lethenteron japonicum. J Exp Biol 210(Pt 21):3821–3829. 10.1242/jeb.007161 17951423 10.1242/jeb.007161

[CR49] King DP, Zhao Y, Sangoram AM et al (1997) Positional cloning of the mouse circadian clock gene. Cell 89(4):641–653. 10.1016/s0092-8674(00)80245-7 9160755 10.1016/s0092-8674(00)80245-7 PMC3815553

[CR50] Kiyoshi K, Kondoh M, Hirunagi K, Korf H (1998) Confocal laser scanning and electron-microscopic analyses of the relationship between VIP-like and GnRH-like-immunoreactive neurons in the lateral septal-preoptic area of the pigeon. Cell Tissue Res 293(1):39–46. 10.1007/s004410051096 9634596 10.1007/s004410051096

[CR51] Klein DC, Weller JL (1970) Indole metabolism in the pineal gland: a circadian rhythm in N-acetyltransferase. Science 169(3950):1093–1095. 10.1126/science.169.3950.1093 4915470 10.1126/science.169.3950.1093

[CR52] Koch M, Dehghani F, Habazettl I, Schomerus C, Korf HW (2006) Cannabinoids attenuate norepinephrine-induced melatonin biosynthesis in the rat pineal gland by reducing arylalkylamine N-acetyltransferase activity without involvement of cannabinoid receptors. J Neurochem 98(1):267–278. 10.1111/j.1471-4159.2006.03873.x 16805813 10.1111/j.1471-4159.2006.03873.x

[CR53] Koch M, Habazettl I, Dehghani F, Korf HW (2008) The rat pineal gland comprises an endocannabinoid system. J Pineal Res 45(4):351–360. 10.1111/j.1600-079X.2008.00597.x 18554250 10.1111/j.1600-079X.2008.00597.x

[CR54] Koch M, Ferreirós N, Geisslinger G, Dehghani F, Korf HW (2015) Rhythmic control of endocannabinoids in the rat pineal gland. Chronobiol Int 32(6):869–874. 10.3109/07420528.2015.1041596 26061461 10.3109/07420528.2015.1041596

[CR55] Korf HW (1974) Acetylcholinesterase-positive neurons in the pineal and parapineal organs of the rainbow trout, Salmo gairdneri (with special reference to the pineal tract). Cell Tissue Res 155(4):475–489. 10.1007/BF00227010 4614911 10.1007/BF00227010

[CR56] Korf HW (1976) Histological, histochemical and electron microscopical studies on the nervous apparatus of the pineal organ in the tiger salamander, Ambystoma tigrinum. Cell Tissue Res 174(4):475–497. 10.1007/BF00232834 1000587 10.1007/BF00232834

[CR57] Korf HW (1994) The pineal organ as a component of the biological clock. Phylogenetic and ontogenetic considerations. Ann N Y Acad Sci 719:13–42. 10.1111/j.1749-6632.1994.tb56818.x 8010588 10.1111/j.1749-6632.1994.tb56818.x

[CR58] Korf HW (2018) Signaling pathways to and from the hypophysial pars tuberalis, an important center for the control of seasonal rhythms. Gen Comp Endocrinol 258:236–243. 10.1016/j.ygcen.2017.05.011 28511899 10.1016/j.ygcen.2017.05.011

[CR59] Korf HW, Fahrenkrug J (1984) Ependymal and neuronal specializations in the lateral ventricle of the Pekin duck. Anas Platyrhynchos Cell Tissue Res 236(1):217–227. 10.1007/BF00216534 6370453 10.1007/BF00216534

[CR60] Korf HW, Stehle J (2002) The circadian system: circuits-cells-clock genes. Cell Tissue Res 309:1–2. 10.1007/s00441-002-0586-z 12171028 10.1007/s00441-002-0586-z

[CR61] Korf HW, von Gall C (2006) Mice, melatonin and the circadian system. Mol Cell Endocrinol 252(1–2):57–68. 10.1016/j.mce.2006.03.005 16644097 10.1016/j.mce.2006.03.005

[CR62] Korf HW, von Gall C (2021) Circadian physiology. In: Pfaff DW, Volkow ND (eds) Neuroscience in the 21st century, 3rd edn. Springer Science+Business Media, New York

[CR63] Korf HW, von Gall C (2024) Mouse models in circadian rhythm and melatonin research. J Pineal Res 76(5):e12986. 10.1111/jpi.12986 38965880 10.1111/jpi.12986

[CR64] Korf HW, Liesner R, Meissl H, Kirk A (1981) Pineal complex of the clawed toad, Xenopus laevis Daud.: structure and function. Cell Tissue Res 216(1):113–30. 10.1007/BF00234548 7226202 10.1007/BF00234548

[CR65] Korf HW, Foster RG, Ekström P, Schalken JJ (1985a) Opsin-like immunoreaction in the retinae and pineal organs of four mammalian species. Cell Tissue Res 242(3):645–648. 10.1007/BF00225432 2934135 10.1007/BF00225432

[CR66] Korf HW, Møller M, Gery I, Zigler JS, Klein DC (1985b) Immunocytochemical demonstration of retinal S-antigen in the pineal organ of four mammalian species. Cell Tissue Res 239(1):81–85. 10.1007/BF00214906 3967288 10.1007/BF00214906

[CR67] Korf HW, Oksche A, Ekström P, Gery I, Zigler JS Jr, Klein DC (1986) Pinealocyte projections into the mammalian brain revealed with S-antigen antiserum. Science 231(4739):735–737. 10.1126/science.3454660 3454660 10.1126/science.3454660

[CR68] Korf B, Rollag MD, Korf HW (1989) Ontogenetic development of S-antigen- and rod-opsin immunoreactions in retinal and pineal photoreceptors of Xenopus laevis in relation to the onset of melatonin-dependent color-change mechanisms. Cell Tissue Res 258(2):319–329. 10.1007/BF00239452 2531037 10.1007/BF00239452

[CR69] Korf HW, Sato T, Oksche A (1990) Complex relationships between the pineal organ and the medial habenular nucleus-pretectal region of the mouse as revealed by S-antigen immunocytochemistry. Cell Tissue Res 261(3):493–500. 10.1007/BF00313528 2245450 10.1007/BF00313528

[CR70] Kramer A (2023) Time for circadian medicine. Acta Physiol (oxf) 238(3):e13984. 10.1111/apha.13984 37211983 10.1111/apha.13984

[CR71] Kramm CM, de Grip WJ, Korf HW (1993) Rod-opsin immunoreaction in the pineal organ of the pigmented mouse does not indicate the presence of a functional photopigment. Cell Tissue Res 274(1):71–78. 10.1007/BF00327987 8242713 10.1007/BF00327987

[CR72] Kuenzel WJ, van Tienhoven A (1982) Nomenclature and location of avian hypothalamic nuclei and associated circumventricular organs. J Comp Neurol 206:293–313 7085935 10.1002/cne.902060309

[CR73] Kume K, Zylka MJ, Sriram S et al (1999) mCRY1 and mCRY2 are essential components of the negative limb of the circadian clock feedback loop. Cell 98(2):193–205 10428031 10.1016/s0092-8674(00)81014-4

[CR74] Lincoln GA, Clarke IJ, Hut RA, Hazlerigg DG (2006) Characterizing a mammalian circannual pacemaker. Science 314(5807):1941–1944. 10.1126/science.1132009 17185605 10.1126/science.1132009

[CR75] Luo W, Li Y, Tang CH, Abruzzi KC, Rodriguez J, Pescatore S, Rosbash M (2012) CLOCK deubiquitylation by USP8 inhibits CLK/CYC transcription in Drosophila. Genes Dev 26(22):2536–2549. 10.1101/gad.200584.112 23154984 10.1101/gad.200584.112 PMC3505823

[CR76] Marchese NA, Ríos MN, Guido ME, Valdez DJ (2024) Three different seasonally expressed opsins are present in the brain of the eared dove, an opportunist breeder. Zoology (jena) 162:126147. 10.1016/j.zool.2024.126147 38277721 10.1016/j.zool.2024.126147

[CR77] Mays JC, Kelly MC, Coon SL et al (2018) Single-cell RNA sequencing of the mammalian pineal gland identifies two pinealocyte subtypes and cell type-specific daily patterns of gene expression. PLoS ONE 13(10):e0205883. 10.1371/journal.pone.0205883 30347410 10.1371/journal.pone.0205883 PMC6197868

[CR78] Meissl H, Ekstrom P (1993) Extraretinal photoreception by pineal systems: a tool for photoperiodic time measurement? Trends Comp Biochem Physiol 1:1223–1240

[CR79] Møller M, van Deurs B, Westergaard E (1978) Vascular permeability to proteins and peptides in the mouse pineal gland. Cell Tissue Res 195(1):1–15. 10.1007/BF00233673 737703 10.1007/BF00233673

[CR80] Morera LP, Díaz NM, Guido ME (2016) Horizontal cells expressing melanopsin x are novel photoreceptors in the avian inner retina. Proc Natl Acad Sci U S A 113(46):13215–13220. 10.1073/pnas.1608901113 27789727 10.1073/pnas.1608901113 PMC5135307

[CR81] Morita Y (1966) Entladungsmuster pinealer Neurone der Regenbogenforelle (Salmo irideus) bei Belichtung des Zwischenhirns [Lead pattern of the pineal neuron of the rainbow trout (Salmo irideus) by illumination of the diencephalon]. Pflugers Arch Gesamte Physiol Menschen Tiere. 289(3):155–67 (German) 5237284

[CR82] Morita Y, Dodt E (1975) Early receptor potential from the pineal photoreceptor. Pflugers Arch 354(3):273–280. 10.1007/BF00584650 1078725 10.1007/BF00584650

[CR83] Nakane Y, Ikegami K, Ono H, Yamamoto N, Yoshida S, Hirunagi K, Ebihara S, Kubo Y, Yoshimura T (2010) A mammalian neural tissue opsin (Opsin 5) is a deep brain photoreceptor in birds. Proc Natl Acad Sci U S A 107(34):15264–15268. 10.1073/pnas.1006393107 20679218 10.1073/pnas.1006393107 PMC2930557

[CR84] Nakao N, Ono H, Yamamura T et al (2008) Thyrotrophin in the pars tuberalis triggers photoperiodic response. Nature 452(7185):317–322. 10.1038/nature06738 18354476 10.1038/nature06738

[CR85] Okano T, Yoshizawa T, Fukada Y (1994) Pinopsin is a chicken pineal photoreceptive molecule. Nature 372(6501):94–97. 10.1038/372094a0 7969427 10.1038/372094a0

[CR86] Oksche A (1984) Evolution of the pineal complex: correlation of structure and function. Ophthalmic Res 16(1–2):88–95. 10.1159/000265300 6728431 10.1159/000265300

[CR87] Oksche A, Hartwig HG (1979) Pineal sense organs–components of photoneuroendocrine systems. Prog Brain Res 52:113–130. 10.1016/S0079-6123(08)62917-9 549077 10.1016/S0079-6123(08)62917-9

[CR88] Oksche A, Kirschstein H (1967) Die Ultrastruktur der Sinneszellen im Pinealorgan von Phoxinus laevis L [Ultrastructure of sensory cells in the pineal body of Phoxinus laevis L]. Z Zellforsch Mikrosk Anat 78(2):151–66 (German) 4872126

[CR89] Oksche A, Kirschstein H (1968) Unterschiedlicher elektronenmikroskopischer Feinbau der Sinneszellen im Parietalauge und im Pinealorgam (Epiphysis cerebri) der Lacertilia. Ein Beitrag zum Epiphysenproblem [Differences in the electron-microscopic structure of the sensory cells in the parietal eye and in the pineal body (epiphysis cerebri) of Lacertilia. A contribution to the problem of the epiphysis]. Z Zellforsch Mikrosk Anat 87(2):159–92 (German) 5707298

[CR90] Oksche A, Kirschstein H (1969) Elektronenmikroskopische Untersuchungen am Pinealorgan von Passerr domesticu [Electron microscopic studies of the pineal organ in Passer domesticus]. Z Zellforsch Mikrosk Anat 102(2):214–41 (German) 5365753

[CR91] Oksche A, Kirschstein H (1971) Weitere elektronenmikroskopische Untersuchungen am Pinealorgan von Phoxinus laevis (Teleostei, Cyprinidae) [Further electron microscopic studies on the pineal organ of Phoxinus laevis (Teleostei, Cyprinidae)]. Z Zellforsch Mikrosk Anat 112(4):572–88 (German) 5542844

[CR92] Oksche A, Vaupel-von Harnack M (1963) Electron microscope studies on the epiphysis cerebri of Rana esculenta L. Z Zellforsch Mikrosk Anat 59:582–614. 10.1007/BF00368731. (German) 13939844 10.1007/BF00368731

[CR93] Oksche A, Vaupel-Von Harnack M (1966) Elektronenmikroskopische Untersuchungen zur Frage der Sinneszellen im Pinealorgan der Vögel [Electron microscopic studies on the problem of sensory cells in the pineal body of birds]. Z Zellforsch Mikrosk Anat 69:41–60 (German) 5973106

[CR94] Oksche A, von Harnack M (1963) Electron microscope studies on the frontal organ of Anura. (On the problem of light receptors). Z Zellforsch Mikrosk Anat 59:239–88 13939843

[CR95] Oksche A, Morita Y, Vaupel-von-Harnack M (1969) Zur Feinstruktur und Funktion des Pinealorgans der Taube (Columba livia) [Ultrastructural and functional studies of the pineal organ in the pigeon (columba livia)]. Z Zellforsch Mikrosk Anat. 102(1):1–30 (German) 4902166

[CR96] Oksche A, Kirschstein H, Kobayashi H, Farner DS (1972) Electron microscopic and experimental studies of the pineal organ in the white-crowned sparrow, Zonotrichia leucophrys gambelii. Z Zellforsch Mikrosk Anat 124(2):247–274. 10.1007/BF00335683 4335080 10.1007/BF00335683

[CR97] Omura Y, Korf HW, Oksche A (1985) Vascular permeability (problem of the blood-brain barrier) in the pineal organ of the rainbow trout, Salmo gairdneri. Cell Tissue Res 239(3):599–610. 10.1007/BF00219238 2580630 10.1007/BF00219238

[CR98] Ono H, Hoshino Y, Yasuo S et al (2008) Involvement of thyrotropin in photoperiodic signal transduction in mice. Proc Natl Acad Sci U S A 105(47):18238–18242. 10.1073/pnas.0808952105 19015516 10.1073/pnas.0808952105 PMC2587639

[CR99] Ostholm T, Brännäs E, van Veen T (1987) The pineal organ is the first differentiated light receptor in the embryonic salmon. Salmo Salar l Cell Tissue Res 249(3):641–646. 10.1007/BF00217336 2959366 10.1007/BF00217336

[CR100] Panda S, Provencio I, Tu DC, Pires SS, Rollag MD, Castrucci AM, Pletcher MT, Sato TK, Wiltshire T, Andahazy M, Kay SA, Van Gelder RN, Hogenesch JB (2003) Melanopsin is required for non-image-forming photic responses in blind mice. Science 301(5632):525–527. 10.1126/science.1086179 12829787 10.1126/science.1086179

[CR101] Patel JS, Woo Y, Draper A, Jansen CS, Carlisle JW, Innominato PF, Lévi FA, Dhabaan L, Master VA, Bilen MA, Khan MK, Lowe MC, Kissick H, Buchwald ZS, Qian DC (2024) Impact of immunotherapy time-of-day infusion on survival and immunologic correlates in patients with metastatic renal cell carcinoma: a multicenter cohort analysis. J Immunother Cancer 12(3):e008011. 10.1136/jitc-2023-008011 38531662 10.1136/jitc-2023-008011 PMC10966813

[CR102] Pérez JH, Tolla E, Bishop VR, Foster RG, Peirson SN, Dunn IC, Meddle SL, Stevenson TJ (2023) Functional inhibition of deep brain non-visual opsins facilitates acute long day induction of reproductive recrudescence in male Japanese quail. Horm Behav 148:105298. 10.1016/j.yhbeh.2022.105298 36621293 10.1016/j.yhbeh.2022.105298

[CR103] Provencio I, Jiang G, De Grip WJ, Hayes WP, Rollag MD (1998) Melanopsin: an opsin in melanophores, brain, and eye. Proc Natl Acad Sci U S A 95(1):340–345. 10.1073/pnas.95.1.340 9419377 10.1073/pnas.95.1.340 PMC18217

[CR104] Provencio I, Rodriguez IR, Jiang G, Hayes WP, Moreira EF, Rollag MD (2000) A novel human opsin in the inner retina. J Neurosci 20(2):600–605. 10.1523/JNEUROSCI.20-02-00600.2000 10632589 10.1523/JNEUROSCI.20-02-00600.2000 PMC6772411

[CR105] Quignon C, Beymer M, Gauthier K, Gauer F, Simonneaux V (2020) Thyroid hormone receptors are required for the melatonin-dependent control of Rfrp gene expression in mice. FASEB J 34(9):12072–12082. 10.1096/fj.202000961R 32776612 10.1096/fj.202000961R

[CR106] Rath MF, Coon SL, Amaral FG, Weller JL, Møller M, Klein DC (2016) Melatonin synthesis: acetylserotonin O-methyltransferase (ASMT) is strongly expressed in a subpopulation of pinealocytes in the male rat pineal gland. Endocrinology 157(5):2028–2040. 10.1210/en.2015-1888 26950199 10.1210/en.2015-1888 PMC4870883

[CR107] Reiter RJ (1991) Melatonin: the chemical expression of darkness. Mol Cell Endocrinol 79(1–3):C153–C158. 10.1016/0303-7207(91)90087-9 1936532 10.1016/0303-7207(91)90087-9

[CR108] Rodríguez EM, Korf HW, Oksche A, Yulis CR, Hein S (1988) Pinealocytes immunoreactive with antisera against secretory glycoproteins of the subcommissural organ: a comparative study. Cell Tissue Res 254(3):469–480. 10.1007/BF00226496 2976614 10.1007/BF00226496

[CR109] Rodriguez EM, Guerra M, Blazquez JL (2024) Roots and routes of neuroendocrinology. Cell Tissue Res (this issue) 10.1007/s00441-024-03928-0 39883141

[CR110] Rosbash M, Bradley S, Kadener S, Li Y, Luo W, Menet JS, Nagoshi E, Palm K, Schoer R, Shang Y, Tang CH (2007) Transcriptional feedback and definition of the circadian pacemaker in Drosophila and animals. Cold Spring Harb Symp Quant Biol 72:75–83. 10.1101/sqb.2007.72.062 10.1101/sqb.2007.72.062 18419264

[CR111] Ruan W, Yuan X, Eltzschig HK (2021) Circadian rhythm as a therapeutic target. Nat Rev Drug Discov 20:287–307. 10.1038/s41573-020-00109-w 33589815 10.1038/s41573-020-00109-w PMC8525418

[CR112] Saldanha CJ, Silverman AJ, Silver R (2001) Direct innervation of GnRH neurons by encephalic photoreceptors in birds. J Biol Rhythms 16(1):39–49. 10.1177/074873040101600105 11220777 10.1177/074873040101600105 PMC3281767

[CR113] Scharrer E (1928) Die Lichtempfindlichkeit blinder Elritzen. Untersuchungen über das Zwischenhirn der Fische I. Z Vergl Physiol 7:1–38

[CR114] Scharrer E (1964) Photo-neuro-endocrine systems: general concepts. Ann N Y Acad Sci 10(117):13–22. 10.1111/j.1749-6632.1964.tb48155.x 10.1111/j.1749-6632.1964.tb48155.x 14196637

[CR115] Schomerus C, Korf HW (2005) Mechanisms regulating melatonin synthesis in the mammalian pineal organ. Ann N Y Acad Sci 1057:372–383. 10.1196/annals.1356.028 16399907 10.1196/annals.1356.028

[CR116] Shearman LP, Zylka MJ, Weaver DR, Kolakowski LF Jr, Reppert SM (1997) Two period homologs: circadian expression and photic regulation in the suprachiasmatic nuclei. Neuron 19(6):1261–1269. 10.1016/s0896-6273(00)80417-1 9427249 10.1016/s0896-6273(00)80417-1

[CR117] Shigeyoshi Y, Taguchi K, Yamamoto S et al (1997) Light-induced resetting of a mammalian circadian clock is associated with rapid induction of the mPer1 transcript. Cell 91(7):1043–1053. 10.1016/s0092-8674(00)80494-8 9428526 10.1016/s0092-8674(00)80494-8

[CR118] Shimmura T, Nakayama T, Shinomiya A, Fukamachi S, Yasugi M, Watanabe E, Shimo T, Senga T, Nishimura T, Tanaka M, Kamei Y, Naruse K, Yoshimura T (2017) Dynamic plasticity in phototransduction regulates seasonal changes in color perception. Nat Commun 8(1):412. 10.1038/s41467-017-00432-8 28871081 10.1038/s41467-017-00432-8 PMC5583187

[CR119] Silver R, Witkovsky P, Horvath P, Alones V, Barnstable CJ, Lehman MN (1988) Coexpression of opsin- and VIP-like-immunoreactivity in CSF-contacting neurons of the avian brain. Cell Tissue Res 253(1):189–198. 10.1007/BF00221754 2970894 10.1007/BF00221754

[CR120] Solessio E, Engbretson GA (1993) Antagonistic chromatic mechanisms in photoreceptors of the parietal eye of lizards. Nature 364:442–445 8332214 10.1038/364442a0

[CR121] Sun ZS, Albrecht U, Zhuchenko O, Bailey J, Eichele G, Lee CC (1997) RIGUI, a putative mammalian ortholog of the Drosophila period gene. Cell 90(6):1003–1011. 10.1016/s0092-8674(00)80366-9 9323128 10.1016/s0092-8674(00)80366-9

[CR122] Tamotsu S, Korf HW, Morita Y, Oksche A (1990) Immunocytochemical localization of serotonin and photoreceptor-specific proteins (rod-opsin, S-antigen) in the pineal complex of the river lamprey, Lampetra japonica, with special reference to photoneuroendocrine cells. Cell Tissue Res 262(2):205–216. 10.1007/BF00309875 2150185 10.1007/BF00309875

[CR123] Tamotsu S, Schomerus C, Stehle JH, Roseboom PH, Korf HW (1995) Norepinephrine-induced phosphorylation of the transcription factor CREB in isolated rat pinealocytes: an immunocytochemical study. Cell Tissue Res 282(2):219–226. 10.1007/BF00319113 8565052 10.1007/BF00319113

[CR124] Tei H, Okamura H, Shigeyoshi Y et al (1997) Circadian oscillation of a mammalian homologue of the Drosophila period gene. Nature 389(6650):512–516. 10.1038/39086 9333243 10.1038/39086

[CR125] Tosini G, Menaker M (1996) Circadian rhythms in cultured mammalian retina. Science 272(5260):419–421. 10.1126/science.272.5260.419 8602533 10.1126/science.272.5260.419

[CR126] Tosini G, Pozdeyev N, Sakamoto K, Iuvone PM (2008) The circadian clock system in the mammalian retina. BioEssays 30(7):624–633. 10.1002/bies.20777 18536031 10.1002/bies.20777 PMC2505342

[CR127] Tosini G, Baba K, Hwang CK, Iuvone PM (2012) Melatonin: an underappreciated player in retinal physiology and pathophysiology. Exp Eye Res 103:82–89. 10.1016/j.exer.2012.08.009 22960156 10.1016/j.exer.2012.08.009 PMC3462291

[CR128] Ueck M (1968) Ultrastruktur des pinealen Sinnesapparates bei einigen Pipidae und Discoglossidae [Ultrastructure of the pineal sensory apparatus in some Pipidae and Discoglossidae]. Z Zellforsch Mikrosk Anat. 92(3):452–76 (German) 4894083

[CR129] van Veen T, Ekström P, Nyberg L, Borg B, Vigh-Teichmann I, Vigh B (1984) Serotonin and opsin immunoreactivities in the developing pineal organ of the three-spined stickleback, Gasterosteus aculeatus L. Cell Tissue Res 237(3):559–564. 10.1007/BF00228440 6237727 10.1007/BF00228440

[CR130] Vigh B, Vigh-Teichmann I (1998) Actual problems of the cerebrospinal fluid-contacting neurons. Microsc Res Tech 41(1):57–83. 10.1002/(SICI)1097-0029(19980401)41:1%3c57::AID-JEMT6%3e3.0.CO;2-R 9550137 10.1002/(SICI)1097-0029(19980401)41:1<57::AID-JEMT6>3.0.CO;2-R

[CR131] Vigh-Teichmann I, Vigh B (1990) Opsin immunocytochemical characterization of different types of photoreceptors in the frog pineal organ. J Pineal Res 8(4):323–333. 10.1111/j.1600-079x.1990.tb00892.x 2144319 10.1111/j.1600-079x.1990.tb00892.x

[CR132] Vigh-Teichmann I, Korf HW, Oksche A, Vigh B (1982) Opsin-immunoreactive outer segments and acetylcholinesterase-positive neurons in the pineal complex of Phoxinus phoxinus (Teleostei, Cyprinidae). Cell Tissue Res 227(2):351–369. 10.1007/BF00210891 6217894 10.1007/BF00210891

[CR133] Vigh-Teichmann I, Korf HW, Nürnberger F, Oksche A, Vigh B, Olsson R (1983) Opsin-immunoreactive outer segments in the pineal and parapineal organs of the lamprey (Lampetra fluviatilis), the eel (Anguilla anguilla), and the rainbow trout (Salmo gairdneri). Cell Tissue Res 230(2):289–307. 10.1007/BF00213806 6221801 10.1007/BF00213806

[CR134] von Frisch K (1911) Beiträge zur Physiologie der Pigmentzellen in der Fischhaut. Pflügers Archiv Ges Physiol 138:319–387

[CR135] Wake K (1973) Acetylcholinesterase-containing nerve cells and their distribution in the pineal organ of the goldfish, Carassius auratus. Z Zellforsch Mikrosk Anat 145(2):287–298. 10.1007/BF00307392 4778596 10.1007/BF00307392

[CR136] Wake K, Ueck M, Oksche A (1974) Acetylcholinesterase-containing nerve cells in the pineal complex and subcommissural area of the frogs, Rana ridibunda and Rana esculenta. Cell Tissue Res 154(4):423–442. 10.1007/BF00219666 4548380 10.1007/BF00219666

[CR137] Wang Y, Narasimamurthy R, Qu M, Shi N, Guo H, Xue Y, Barker N (2024) Circadian regulation of cancer stem cells and the tumor microenvironment during metastasis. Nat Cancer 5:546–556. 10.1038/s43018-024-00759-4 10.1038/s43018-024-00759-4 38654103

[CR138] Wang G, Wingfield JC (2011) Immunocytochemical study of rhodopsin-containing putative encephalic photoreceptors in house sparrow. Passer Domesticus Gen Comp Endocrinol 170(3):589–596. 10.1016/j.ygcen.2010.11.014 21118688 10.1016/j.ygcen.2010.11.014

[CR139] Wyart C, Carbo-Tano M, Orts-Del’Immagine Y, Orts-Delimmagine A, Böhm UL (2023) Cerebrospinal fluid-contacting neurons: multimodal cells with diverse roles in the CNS. Nat Rev Neurosci 24(9):540–556. 10.1038/s41583-023-00723-8 37558908 10.1038/s41583-023-00723-8

[CR140] Yamaguchi S, Kobayashi M, Mitsui S et al (2001) View of a mouse clock gene ticking. Nature 409(6821):684. 10.1038/35055628 11217850 10.1038/35055628

[CR141] Yasuo S, Korf HW (2011) The hypophysial pars tuberalis transduces photoperiodic signals via multiple pathways and messenger molecules. Gen Comp Endocrinol 172(1):15–22. 10.1016/j.ygcen.2010.11.006 21078321 10.1016/j.ygcen.2010.11.006

[CR142] Yasuo S, Yoshimura T, Ebihara S, Korf HW (2009) Melatonin transmits photoperiodic signals through the MT1 melatonin receptor. J Neurosci 29(9):2885–2889. 10.1523/JNEUROSCI.0145-09.2009 19261884 10.1523/JNEUROSCI.0145-09.2009 PMC6666200

[CR143] Yasuo S, Koch M, Schmidt H, Ziebell S, Bojunga J, Geisslinger G, Korf HW (2010) An endocannabinoid system is localized to the hypophysial pars tuberalis of Syrian hamsters and responds to photoperiodic changes. Cell Tissue Res 340(1):127–136. 10.1007/s00441-010-0930-7 20165884 10.1007/s00441-010-0930-7

[CR144] Yasuo S, Unfried C, Kettner M, Geisslinger G, Korf HW (2010) Localization of an endocannabinoid system in the hypophysial pars tuberalis and pars distalis of man. Cell Tissue Res 342(2):273–281. 10.1007/s00441-010-1066-5 20957495 10.1007/s00441-010-1066-5

[CR145] Yoshimura T, Yasuo S, Watanabe M et al (2003) Light-induced hormone conversion of T4 to T3 regulates photoperiodic response of gonads in birds. Nature 426(6963):178–181. 10.1038/nature02117 14614506 10.1038/nature02117

[CR146] Young MW (2018) Time Travels: A 40-year journey from Drosophila’s clock mutants to human circadian disorders (Nobel lecture). Angew Chem Int Ed Engl 57(36):11532–11539. 10.1002/anie.201803337 30003624 10.1002/anie.201803337

[CR147] Zeng Y, Guo Z, Wu M, Chen F, Chen L (2024) Circadian rhythm regulates the function of immune cells and participates in the development of tumors. Cell Death Discov 10(1):199. 10.1038/s41420-024-01960-1 38678017 10.1038/s41420-024-01960-1 PMC11055927

[CR148] Zhao H, Jiang J, Wang G, Le C, Wingfield JC (2018) Daily, circadian and seasonal changes of rhodopsin-like encephalic photoreceptor and its involvement in mediating photoperiodic responses of Gambel’s white-crowned sparrow, Zonotrichia leucophrys gambelii. Brain Res 15(1687):104–116. 10.1016/j.brainres.2018.02.048 10.1016/j.brainres.2018.02.048 29510141

